# Exact SER Analysis of Partial-CSI-Based SWIPT OAF Relaying over Rayleigh Fading Channels and Insights from a Generalized Non-SWIPT OAF Approximation

**DOI:** 10.3390/s25154872

**Published:** 2025-08-07

**Authors:** Kyunbyoung Ko, Seokil Song

**Affiliations:** 1 Department of Electronics Engineering, Korea National University of Transportation, 50 Daehak-ro, Chungju-si 27469, Republic of Korea; kbko@ut.ac.kr; 2 Department of Computer Engineering, Korea National University of Transportation, 50 Daehak-ro, Chungju-si 27469, Republic of Korea

**Keywords:** SER, SWIPT, OAF relay, PS, MGF, CSI

## Abstract

This paper investigates the error rate performance of simultaneous wireless information and power transfer (SWIPT) systems employing opportunistic amplify-and-forward (OAF) relaying under Rayleigh fading conditions. To support both data forwarding and energy harvesting at relays, a power splitting (PS) mechanism is applied. We derive exact and asymptotic symbol error rate (SER) expressions using moment-generating function (MGF) methods, providing analytical insights into how the power splitting ratio ρ and the quality of source–relay (SR) and relay–destination (RD) links jointly affect system behavior. Additionally, we propose a novel approximation that interprets the SWIPT-OAF configuration as an equivalent non-SWIPT OAF model. This enables tractable performance analysis while preserving key diversity characteristics. The framework is extended to include scenarios with partial channel state information (CSI) and $N_{\mathrm{th}}$ best relay selection, addressing practical concerns such as limited relay availability and imperfect decision-making. Extensive simulations validate the theoretical analysis and demonstrate the robustness of the proposed approach under a wide range of signal-to-noise ratio (SNR) and channel conditions. These findings contribute to a flexible and scalable design strategy for SWIPT-OAF relay systems, making them suitable for deployment in emerging wireless sensor and internet of things (IoT) networks.

## 1. Introduction

The increasing demand for sustainable and energy-efficient wireless networks has propelled significant interest in simultaneous wireless information and power transfer (SWIPT), where wireless devices harvest energy from radio frequency (RF) signals while simultaneously receiving data [1,2,3]. To leverage this capability, SWIPT has been widely integrated into cooperative relaying systems, enabling relays to utilize harvested energy to forward information, thereby enhancing both coverage and network lifetime [4,5,6,7,8,9,10].

Among various receiver architectures, power splitting (PS) has emerged as a practical technique, allowing simultaneous energy harvesting (EH) and information processing (IP) by dividing the received signal power into separate streams [5,7]. Despite its practicality, PS introduces additional complexity into the analysis of key performance metrics, such as symbol error rate (SER) and bit error rate (BER), which are crucial for system design and optimization.

Recently, SWIPT amplify-and-forward (AF) relay systems have attracted considerable attention due to their potential for enhancing both energy efficiency and spectral efficiency in diverse network architectures [11,12,13,14,15,16,17,18,19]. Various studies have explored power allocation strategies [11], relay selection under energy constraints [7,12], and advanced receiver designs that jointly optimize information decoding and energy harvesting [13]. Additionally, challenges related to residual hardware impairments [8] and nonlinear EH models [15] have been investigated to improve the practical feasibility of SWIPT AF systems. However, the majority of existing works focus on single-relay scenarios, perfect channel knowledge, or simplified performance bounds, leaving significant gaps in understanding SWIPT-enabled opportunistic AF (OAF) relaying under partial channel state information (CSI) [20].

For conventional non-SWIPT relay networks, extensive analytical results exist for various relaying protocols, channel models, and diversity techniques [21,22,23,24,25,26]. In particular, OAF relaying, where only the best relay forwards the source signal, has been recognized as an efficient means of achieving a balance between diversity gains and spectral efficiency [24,25,27]. Such relay selection strategies help reduce the number of required orthogonal channels, mitigating the loss in spectral efficiency typical of regular cooperative diversity systems [22,28,29].

In parallel with analytical studies, several recent works have focused on practical applications of SWIPT-enabled wireless systems, particularly in the context of internet of things (IoT) and uncrewed aerial vehicle (UAV)-assisted networks. For example, the authors in [30] analyzed the outage performance of a UAV-assisted multiple-input–single-output (MISO)-nonorthogonal multiple access (NOMA) downlink agricultural-IoT system with SWIPT and transmit antenna selection enhancement. Similarly, Lin et al. [31] proposed and evaluated a lossy forwarding-based SWIPT relaying scheme with quality of service (QoS) guarantees, focusing on outage behavior under practical constraints.

While these studies contribute to understanding SWIPT in application-specific domains such as agricultural IoT and lossy relaying, they primarily address outage probability, NOMA transmission, or application-layer concerns. In contrast, the present work targets a fundamentally different aspect: an exact and asymptotic SER analysis of partial-CSI-based OAF relaying systems over Rayleigh fading channels. Furthermore, we introduce a generalized non-SWIPT OAF approximation, enabling unified and tractable performance analysis not addressed in previous works. Therefore, our study complements the aforementioned research by exploring performance at the physical layer, offering deeper insights into error rate behavior under both partial CSI and energy harvesting constraints.

Nevertheless, practical constraints may hinder the selection of the best relay due to scheduling, load balancing, or errors in relay selection, prompting interest in analyzing systems employing the $N_{\mathrm{th}}$ best relay [29,32,33]. For instance, the exact SER performance for the $N_{\mathrm{th}}$ best opportunistic AF relay systems was analyzed in [32], while Ko et al. [33] investigated outage probability and channel capacity under the independent and non-identically distributed (INID) Rayleigh fading. Approximating multi-hop relaying as an equivalent dual-hop model was proposed in [34], and the impact of channel estimation errors on opportunistic AF relays was addressed in [35]. Furthermore, Ko et al. [36] introduced an analytical framework interpreting SWIPT AF relay systems as equivalent non-SWIPT AF relays, facilitating tractable BER analysis and PS factor optimization.

Despite these valuable contributions, research on SWIPT-enabled OAF relaying under partial CSI remains limited. In particular, deriving exact SER expressions for SWIPT OAF relay networks over Rayleigh fading, while considering practical scenarios such as $N_{\mathrm{th}}$ best relay selection, poses significant analytical challenges due to the dual impact of relay selection diversity and power harvesting constraints. Furthermore, there is a lack of studies that propose practical operational protocols or deployment scenarios where SWIPT OAF relaying could be effectively implemented.

Motivated by our prior work in [36], where SWIPT AF relaying was successfully approximated as a generalized non-SWIPT AF relay to simplify performance analysis, this paper extends the concept to opportunistic AF relaying. In addition to providing a theoretical analysis, we propose a practically viable operational scenario that supports SWIPT OAF relaying. For instance, SWIPT OAF relaying can be employed in wireless sensor networks or IoT environments, where relays harvest energy from nearby transmissions and selectively forward information based on partial CSI. Such a protocol can significantly reduce system complexity while preserving diversity benefits.

The main contributions of this paper are summarized as follows:

1. Operational Protocol Proposal: A practical protocol and operational scenario are proposed for efficient SWIPT OAF relaying under partial-CSI (P-CSI) and energy harvesting constraints.
2. Exact SER Analysis: Exact closed-form expressions for the SER of P-CSI-based SWIPT OAF relaying systems over Rayleigh fading channels are derived using moment-generating function (MGF)-based techniques.
3. Generalized Non-SWIPT OAF Approximation: An analytical approximation modeling the SWIPT OAF relay as a generalized non-SWIPT OAF relay is introduced, enabling a unified and tractable performance analysis.
4. Analysis of $N_{\mathrm{th}}$ Best Relay Selection: The framework incorporates scenarios involving $N_{\mathrm{th}}$ best relay selection, addressing practical issues such as relay unavailability or selection inaccuracies.
5. Asymptotic Performance Insights: Asymptotic SER expressions in the high signal-to-noise ratio (SNR) regime are derived, providing insights into the harmonic mean behavior of end-to-end SNR and the influence of PS ratios.
6. Simulation Validation: Extensive simulations confirm the analytical results and quantify performance trade-offs associated with PS factors, relay selection strategies, and partial CSI.

The remainder of this paper is organized as follows. Section 2 describes the system model. Section 3 presents the exact SER derivation and the generalized non-SWIPT OAF interpretation based on P-CSI from the source-to-relay (SR) link. Section 4 analyzes the error rate performance under P-CSI from the relay-to-destination (RD) link. Section 5 provides numerical and simulation results. Finally, Section 6 concludes the paper.

## 2. SWIPT OAF Relaying Systems

We consider a point-to-point communication system in which cooperative relays assist data transmission between the source and the destination. Each relay operates in AF half-duplex mode, and all nodes are equipped with a single antenna. The relay nodes do not have dedicated power supplies and instead harvest energy from the source’s RF signals. To simultaneously perform EH and information processing at the relay, a PS receiver is employed, which divides the received signal power between the EH circuit and the information processing unit [1,2,36].

### 2.1. SWIPT Relaying System Model

**Figure 1:**
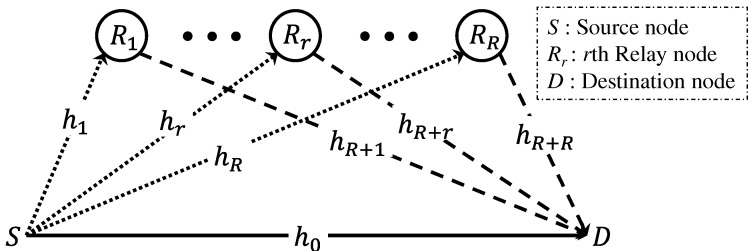
Block diagram of a SWIPT relaying system with *R* relay nodes.

To describe SWIPT-based opportunistic AF relaying systems, we first introduce the general SWIPT AF relaying scheme and present the corresponding signal models. Let us consider a SWIPT relaying system with *R* relay nodes of Figure 1. The channel gains for the source-to-destination (SD), SR, and RD links are denoted by $h_{0}$, ${\left\{h_{r}\right\}}_{r=1}^{R}$, and ${\left\{h_{R+r}\right\}}_{r=1}^{R}$, respectively. These are modeled as mutually independent Rayleigh-distributed random variables.

Due to the half-duplex nature of the relays, communication occurs over two time slots. In the first time slot, the source broadcasts a signal $x_{s}$ satisfying $E\left[x_{s}\right]=0$ and $E\left[{\left|x_{s}\right|}^{2}\right]=P_{s}$ to the relays and the destination. Here, $E\left[\cdot \right]$ denotes the statistical expectation. Accordingly, the received signals at the *r*th relay and the destination are given by

(1) $$\begin{array}{cc} y_{r} & =h_{r}x_{s}+n_{r}\\ y_{0} & =h_{0}x_{s}+n_{0} \end{array}$$

where $n_{r}$ and $n_{0}$ are independent additive white Gaussian noise (AWGN) terms at the *r*th relay and the destination, respectively, with $E\left[n_{r}\right]=E\left[n_{0}\right]=0$ and $E\left[{\left|n_{r}\right|}^{2}\right]=E\left[{\left|n_{0}\right|}^{2}\right]=\sigma^{2}$.

The power splitter at the *r*th relay divides the received signal $y_{r}$ into two components: $y_{r}^{E}$ for energy harvesting and $y_{r}^{I}$ for information processing. By introducing a power splitting ratio $\rho_{r}$ with $0<\rho_{r}<1$ [2], we express

(2) $$\begin{array}{cc} y_{r}^{E} & \;=\sqrt{\rho_{r}}y_{r}=\sqrt{\rho_{r}}\left(h_{r}x_{s}+n_{r}\right) \end{array}$$

and then, the total harvested energy at the *r*th relay during the first time slot is given by $E_{r}^{h}\;=E\left[{\left|y_{r}^{E}\right|}^{2}\right]=\eta \rho_{r}P_{s}{\left|h_{r}\right|}^{2}T$ where $\eta \in \left(0,1\right]$ denotes the energy conversion efficiency, and *T* is the time duration of the first time slot. Since the AF relay uses an equal duration for receiving and forwarding, the available transmit power at the *r*th relay in the second phase becomes

(3) $$\begin{array}{cc} P_{r} & \;=\eta \rho_{r}P_{s}{\left|h_{r}\right|}^{2}. \end{array}$$

Meanwhile, the signal for information processing is expressed as

(4) $$\begin{array}{cc} y_{r}^{I}\; & =\sqrt{1-\rho_{r}}y_{r}+n_{c_{r}}=\sqrt{1-\rho_{r}}h_{r}x_{s}+n_{R_{r}} \end{array}$$

where $n_{R_{r}}=\sqrt{1-\rho_{r}}n_{r}+n_{c_{r}}$ and $n_{c_{r}}$ denotes the noise introduced during the RF band-to-baseband conversion. It is assumed that $n_{r}$ and $n_{c_{r}}$ are mutually independent, zero-mean, and have equal power, i.e., $E\left[n_{c_{r}}\right]=E\left[n_{R_{r}}\right]=0$, $E\left[{\left|n_{c_{r}}\right|}^{2}\right]=\sigma^{2}$, and $\sigma_{R_{r}}^{2}=E\left[{\left|n_{R_{r}}\right|}^{2}\right]=\left(2-\rho_{r}\right)\sigma^{2}$.

In the second time slot, the *r*th relay forwards $y_{r}^{I}$ to the destination using the instantaneous power budget $P_{r}$. The transmitted signal is given by

(5) $$\begin{array}{cc} x_{r} & \;=\kappa_{r}y_{r}^{I} \end{array}$$

where $\kappa_{r}=\sqrt{\frac{\eta \rho_{r}P_{s}{\left|h_{r}\right|}^{2}}{\left(1-\rho_{r}\right){\left|h_{r}\right|}^{2}P_{s}+\sigma_{R_{r}}^{2}}}$ is the amplifying gain. Then, the destination receives $y_{R+r}$ from the *r*th relay as

(6) $$\begin{array}{cc} y_{R+r}\; & =h_{R+r}x_{r}+n_{R+r}\\ \; & =h_{R+r}\kappa_{r}\sqrt{1-\rho_{r}}h_{r}x_{s}+h_{R+r}\kappa_{r}\left(\sqrt{1-\rho_{r}}n_{R_{r}}+n_{c_{r}}\right)+n_{R+r} \end{array}$$

where $n_{R+r}$ denotes the AWGN at the destination during the second time slot, with $E\left[n_{R+r}\right]=0$ and $E\left[{\left|n_{R+r}\right|}^{2}\right]=\sigma^{2}$. Note that all noise components $\left\{n_{0},n_{r},n_{c_{r}},n_{R+r}\right\}$ are assumed to be mutually independent.

#### Indirect (SRD) Link SNR

From $y_{R+r}$ of (6), the indirect (i.e., source–relay–destination (SRD)) link SNR at the *r*th relay is given by

(7) $$\begin{array}{cc} {\mathrm{SNR}}_{{\mathrm{id}}_{r}}\; & =\frac{P_{s}\kappa^{2}\left(1-\rho_{r}\right){\left|h_{r}\right|}^{2}{\left|h_{R+r}\right|}^{2}}{\kappa^{2}{\left|h_{R+r}\right|}^{2}\sigma_{R_{r}}^{2}+\sigma^{2}}. \end{array}$$

At high SNR (i.e., $P_{s}\gg \sigma^{2}$) [2,36], this can be approximated as

(8) $$\begin{array}{cc} {\mathrm{SNR}}_{{\mathrm{id}}_{r}}≃\gamma_{{\mathrm{id}}_{r}}\; & =\frac{\gamma_{r}\beta_{r}}{\beta_{r}+1} \end{array}$$

where

(9) $$\begin{array}{cc} \gamma_{r} & =\frac{\left(1-\rho_{r}\right)P_{s}{\left|h_{r}\right|}^{2}}{\sigma_{R_{r}}^{2}}=\gamma_{{\mathrm{sr}}_{r}}\\ \gamma_{{\mathrm{rd}}_{r}} & =\frac{\eta \rho_{r}P_{s}{\left|h_{R+r}\right|}^{2}}{\sigma^{2}}\\ \beta_{r}\; & =\gamma_{{\mathrm{rd}}_{r}}\frac{\Omega_{r}}{{\bar{\gamma }}_{r}} \end{array}$$

and

(10) $$\begin{array}{cc} {\bar{\gamma }}_{r}\; & =\left(1-\rho_{r}\right)P_{s}\Omega_{{\mathrm{sr}}_{r}}/\sigma_{R_{r}}^{2}={\bar{\gamma }}_{{\mathrm{sr}}_{r}}\\ {\bar{\gamma }}_{{\mathrm{rd}}_{r}}\; & =\eta \rho_{r}P_{s}\Omega_{{\mathrm{rd}}_{r}}/\sigma^{2}\\ {\bar{\beta }}_{r}\; & ={\bar{\gamma }}_{{\mathrm{rd}}_{r}}\Omega_{{\mathrm{sr}}_{r}}/{\bar{\gamma }}_{{\mathrm{sr}}_{r}}, \end{array}$$

$E\left[{\left|h_{r}\right|}^{2}\right]=\Omega_{r}=\Omega_{{\mathrm{sr}}_{r}}$, and $E\left[{\left|h_{R+r}\right|}^{2}\right]=\Omega_{R+r}=\Omega_{{\mathrm{rd}}_{r}}$. Note that $\gamma_{r}$$\left(=\gamma_{{\mathrm{sr}}_{r}}\right)$ and $\gamma_{{\mathrm{rd}}_{r}}$ represent the SR and RD link SNRs, respectively.

### 2.2. SWIPT Relay Selection Scenario for SRD $\left(S\to R\to D\right)$ Link

**Figure 2:**
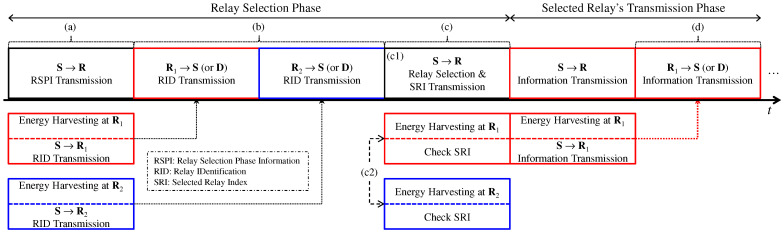
Block diagram of the relay selection process utilizing P-CSI based on SR link (R=2).

In this section, we present a SWIPT relay selection method based on partial CSIs of SR link channels. Figure 2 illustrates the block diagram of the relay selection process for the case of R=2 relays. In Figure 2, the block, red, and blue boxes represent the operations of the source, the first SWIPT relay, and the second SWIPT relay nodes, respectively. The relay selection is performed at the source node during the initialization phase of the dual-hop multi-relay SWIPT system.

The relay selection is executed at the source node during the relay selection phase (RSP) depicted in Figure 2. As shown, the process consists of three subphases: RSP(a), RSP(b), and RSP(c). It is noted that, as shown in Figure 2, RSP(c) is composed of (c1) and (c2).

(a) Source node: Broadcasts basic information (i.e., relay selection phase information, RSPI) to all SWIPT relays.
(b) Each SWIPT relay: Performs EH and responds to both the source and destination nodes by transmitting its relay identification (RID) (e.g., an assigned Walsh–Hadamard code [35]).
(c1) Source node: Computes the instantaneous SR link SNR, selects the best relay, and broadcasts the selected relay index (SRI) to all relays.
(c2) Each SWIPT relay: Receives the SRI and compares it with its own RID.
(d) Selected relay: Participates in the AF transmission phase.

In this paper, we assume that all relay nodes employ an initial PS factor of ${\left.\rho_{r}\right|}_{r=1}^{R}=\rho_{0}$ before PS optimization is applied. Accordingly, the instantaneous link SNRs in (9) can be expressed as

(11) $$\begin{array}{cc} \gamma_{r}^{0} & =\frac{\left(1-\rho_{0}\right)P_{s}{\left|h_{r}\right|}^{2}}{\sigma_{R_{r}}^{2}}=\gamma_{{\mathrm{sr}}_{r}}^{0}\\ \gamma_{{\mathrm{rd}}_{r}}^{0} & =\frac{\eta \rho_{0}P_{s}{\left|h_{R+r}\right|}^{2}}{\sigma^{2}}\\ \beta_{r}^{0}\; & =\gamma_{{\mathrm{rd}}_{r}}^{0}/\frac{\left(1-\rho_{0}\right)P_{s}}{\sigma_{R_{r}}^{2}}\\ \sigma_{R_{r}}^{2} & =\left(2-\rho_{0}\right)\sigma^{2}. \end{array}$$

#### 2.2.1. RSP(a): S→R Subphase

In the relay selection phase (a), the received signal and the SR link SNR at the *r*th relay can be expressed as

(12) $$\begin{array}{cc} y_{{\mathrm{sr}}_{r}} & \;=h_{r}x_{s}+n_{{\mathrm{sr}}_{r}} \end{array}$$

where $n_{{\mathrm{sr}}_{r}}$ denotes the AWGN at the *r*th relay node with $E\left[n_{{\mathrm{sr}}_{r}}\right]=0$ and $E\left[{\left|n_{{\mathrm{sr}}_{r}}\right|}^{2}\right]=\sigma^{2}$.

#### 2.2.2. RSP(b): S→R (Non-AF) →S Subphase

Each SWIPT relay node responds to both the source and the destination nodes. In this subphase, each relay transmits its RID information $x_{r}^{\mathrm{RID}}$ (e.g., an assigned Walsh–-Hadamard code [35]). During this phase, the received signal at each relay is not forwarded but used solely for energy harvesting. From (12), the transmit symbol at the *r*th relay can be expressed as $x_{{\mathrm{rs}}_{r}}=\kappa_{{\mathrm{rs}}_{r}}\sqrt{1-\rho_{0}}x_{r}^{\mathrm{RID}}$ where $\kappa_{{\mathrm{rs}}_{r}}=\sqrt{\frac{\eta \rho_{0}P_{s}{\left|h_{r}\right|}^{2}}{1-\rho_{0}}}$, $E\left[x_{r}^{\mathrm{RID}}\right]=0$, and $E\left[{\left|x_{r}^{\mathrm{RID}}\right|}^{2}\right]=1$.

At the source node, the received signal can be expressed as

(13) $$\begin{array}{cc} y_{{\mathrm{srs}}_{r}}\; & =h_{r}x_{{\mathrm{rs}}_{r}}+n_{{\mathrm{rs}}_{r}}=h_{r}\kappa_{{\mathrm{rs}}_{r}}\sqrt{1-\rho_{0}}x_{r}^{\mathrm{RID}}+n_{{\mathrm{rs}}_{r}} \end{array}$$

where $n_{{\mathrm{rs}}_{r}}$ denotes the AWGN at the source node with $E\left[n_{{\mathrm{rs}}_{r}}\right]=0$ and $E\left[{\left|n_{{\mathrm{rs}}_{r}}\right|}^{2}\right]=\sigma^{2}$.

#### 2.2.3. RSP(c1): CSI at Source Node

At the source node, the received SNR can be estimated from (13) as

(14) $$\begin{array}{cc} {\hat{\gamma }}_{{\mathrm{srs}}_{r}}\; & =\frac{\eta \rho_{0}P_{s}{\left|h_{r}\right|}^{4}}{\sigma^{2}}. \end{array}$$

From (13), to coherently detect the RID information, the source node must estimate the channel gain. The perfectly estimated channel coefficient is then expressed as

(15) $${\hat{h}}_{{\mathrm{srs}}_{r}}=\sqrt{\eta \rho_{0}P_{s}}{\left|h_{r}\right|}^{2}.$$

Note that the source node is aware of the SWIPT relay index *r*, as well as the parameters η and $P_{s}$. $P_{s}$ is both predefined and known to all nodes. The parameter η can be established through field measurements or dictated by hardware specifications.

From (11) and (13), it can be observed that

(16) $$\begin{array}{cc} \gamma_{r}^{0} & =\frac{\left(1-\rho_{0}\right)P_{s}{\left|h_{r}\right|}^{2}}{\sigma_{R_{r}}^{2}}\propto \sqrt{{\hat{\gamma }}_{{\mathrm{srs}}_{r}}}\propto {\hat{h}}_{{\mathrm{srs}}_{r}}\propto {\left|h_{r}\right|}^{2}. \end{array}$$

Therefore, the source node can select the best relay based on ${\left\{{\hat{h}}_{{\mathrm{srs}}_{r}}\right\}}_{r=1}^{R}$ in (16). From (15), (16), and ${\left.\gamma_{r}^{0}\right|}_{r=1}^{R}$ in (11), the relay selection algorithm can be expressed as

(17) $$\begin{array}{cc} i\; & =\arg\max_{r\in \{1,2,\cdots ,R\}}\left\{{\hat{h}}_{{\mathrm{srs}}_{r}}\right\}=\arg\max_{r\in \{1,2,\cdots ,R\}}\left\{{\left|h_{r}\right|}^{2}\right\}=\arg\max_{r\in \{1,2,\cdots ,R\}}\left\{\gamma_{r}^{0}\right\}. \end{array}$$

Note that the relay selection process is performed at the source node prior to the PS optimization at the selected relay. Accordingly, in (17), $\rho_{r}=\rho_{0}$ is used as a predetermined initial value.

#### 2.2.4. RSP(c2) & (d): Source Transmits the Selected Relay Index

Each SWIPT relay node receives the SRI and compares it with its own RID. Only the relay whose RID matches the received SRI proceeds to the SWIPT AF retransmission mode (i.e., the transmission phase of the selected relay). Note that, in this paper, each relay adopts a self-optimization scheme designed to minimize the asymptotic error rate. As proposed in [36], the optimal PS factor is determined to minimize the asymptotic error rate and is given by

(18) $$\begin{array}{cc} \rho_{{\mathrm{opt}}_{r}}\; & =\arg\min_{0<\rho_{r}<1}P_{B,\mathrm{id}}^{\mathrm{Asym}}\left({\bar{\gamma }}_{r},{\bar{\beta }}_{r}\right) \end{array}$$

where $P_{B,\mathrm{id}}^{\mathrm{Asym}}\left({\bar{\gamma }}_{r},{\bar{\beta }}_{r}\right)$ is presented as the approximated form in (A9). Note that ${\bar{\gamma }}_{r}$ and ${\bar{\beta }}_{r}$ are functions of $\rho_{r}$. From (10), it can be observed that

$$\begin{array}{cc} {\bar{\gamma }}_{r}\; & =\frac{\left(1-\rho_{r}\right)P_{s}\Omega_{r}}{\sigma_{R_{r}}^{2}}\\ {\bar{\beta }}_{r}{\bar{\gamma }}_{r}\; & =\frac{\eta \rho_{r}P_{s}\Omega_{R+r}\Omega_{r}}{\sigma^{2}}={\bar{\gamma }}_{{\mathrm{rd}}_{r}}\Omega_{r}. \end{array}$$

These expressions clearly show the dependence of ${\bar{\gamma }}_{r}$ and ${\bar{\beta }}_{r}$ on the PS factor $\rho_{r}$. Note that each SWIPT relay node is assumed to have knowledge of η, $\rho_{r}$, and $P_{s}$. The parameter η can be predetermined through field measurements or specified by hardware constraints. $P_{s}$ is assumed to be initial value shared and known by all nodes in the network.

Each SWIPT relay node is capable of computing the amplifying gains for both the ‘S→R Phase’ and the ‘D→R Phase’. During the ‘S→R Phase’, the relay calculates the available transmit power as $P_{{\mathrm{sr}}_{r}}=\eta \rho_{r}P_{s}{\left|h_{r}\right|}^{2}$. Similarly, in the ‘D→R Phase’, the available transmit power is computed as $P_{{\mathrm{dr}}_{r}}=\eta \rho_{r}P_{d}{\left|h_{R+r}\right|}^{2}$ where $P_{d}$ is the destination transmitting symbol power. From the expected values $E\left[P_{{\mathrm{sr}}_{r}}\right]$ and $E\left[P_{{\mathrm{dr}}_{r}}\right]$, the relay node can estimate the channel statistics as $E\left[{\left|h_{r}\right|}^{2}\right]=\Omega_{r}$ and $E\left[{\left|h_{R+r}\right|}^{2}\right]=\Omega_{R+r}$, respectively. To address the computational complexity associated with optimizing $\rho_{r}$, a precomputed vector table can be constructed as a function of ${\bar{\gamma }}_{r}$, $\Omega_{r}$, and ${\bar{\gamma }}_{{\mathrm{rd}}_{r}}$. The robustness of the optimized power splitting factor has been verified through simulations and numerical evaluations, as demonstrated in [36].

Therefore, it is concluded that implementing the optimal $\rho_{r}$ using a lookup table is practical and efficient. Consequently, each SWIPT relay node can autonomously optimize the power splitting factor $\rho_{r}$ during the initial setup process by utilizing both the ‘S→R Phase’ and the ‘D→R Phase’.

## 3. Performance Analysis for the SWIPT OAF Relaying System: P-CSI Based on SR Link

The performance of the OAF technique degrades when the optimal relay is not selected. Accordingly, this paper investigates the performance associated with selecting the *N*th best relay. In this section, the selection probability of the *i*th relay from ${\left.\gamma_{r}^{0}\right|}_{r=1}^{R}$ is first derived. Next, the average BER for SWIPT OAF schemes is presented, followed by asymptotic BER expressions.

### 3.1. Nth Best Selection Probability

To derive the exact ASER expression for the SWIPT OAF relay system, we first consider the selection algorithm based on P-CSI. Note that, although the relay selection method described in Section 2.2 is based on ${\left\{{\hat{h}}_{{\mathrm{srs}}_{r}}\right\}}_{r=1}^{R}$ in (16), the OAF performance analysis is conducted using ${\left\{\gamma_{r}^{0}\right\}}_{r=1}^{R}$ from (11). The rationale for this approach is provided in (17). Consequently, based on (17) and ${\left\{\gamma_{r}^{0}\right\}}_{r=1}^{R}$ in (11), the relay selection algorithm can be expressed as

(19) $$\begin{array}{cc} i & \;=\arg\left(N_{\mathrm{th}}\max{\left\{\gamma_{r}^{0}\right\}}_{r=1}^{R}\right). \end{array}$$

In addition, each relay node can perform PS optimization independently. Therefore, the random variable pair $\left\{\gamma_{i},\beta_{i}\right\}$ with the optimized $\rho_{i}$ is selected during the transmission phase of the selected relay shown in Figure 2.

#### 3.1.1. Relay Selection Probability for the *i*th Relay

The selection in (19) corresponds to the problem of choosing the $N_{\mathrm{th}}$ maximum value among *R* independent RVs. Each of the *R* relays can be selected as the $N_{\mathrm{th}}$ best relay. Using (A23), (A24), and (A25), the selection probability of the RV $\gamma_{i}^{0}$ can be expressed as

(20) pγi0x=∑j=1R−1N−1∑k=0R−N(−1)k∑l=1R−Nkexp−xBij,k,l

where

$$B_{i}^{j,k,l}=\sum_{m=1}^{N-1}1/{\bar{\gamma }}_{\lambda_{i,m}^{N-1,j}}^{0}+\sum_{m=1}^{k}1/{\bar{\gamma }}_{{\left(\lambda_{i}^{\bar{N-1},j}\right)}_{m}^{k,l}}^{0},$$

${\left.\sum_{m=1}^{k}\left(\cdot \right)\right|}_{k=0}=0$, and ${\left.{\bar{\gamma }}_{r}^{0}\right|}_{r=1}^{R}$ of (11). The detailed derivation of (20) is provided in Appendix B.

#### 3.1.2. Joint PDF

From (19) and (20), the joint probability density function (PDF) of selecting the $N_{\mathrm{th}}$ random variable pair from ${\left\{\gamma_{i}^{0},\beta_{i}\right\}}_{i=1}^{R}$ is given by [32,33]

(21) $$\begin{array}{cc} f_{\gamma_{N_{\max}}^{0},\beta_{N_{\max}}}\left(x,y\right)\; & =\sum_{i=1}^{R}p_{\gamma_{i}^{0}}\left(x\right)f_{\gamma_{i}^{0},\beta_{i}}\left(x,y\right)\\ \; & =\underset{i,j,k,l}{\underset{︸}{\sum \cdots \sum }}\frac{{\bar{\gamma }}_{i}^{\prime }{\left(-1\right)}^{k}}{{\bar{\gamma }}_{i}^{0}}\frac{1}{{\bar{\gamma }}_{i}^{\prime }}e^{-x/{\bar{\gamma }}_{i}^{\prime }}\frac{1}{{\bar{\beta }}_{i}}e^{-y/{\bar{\beta }}_{i}}u\left(x\right)u\left(y\right) \end{array}$$

where $1/{\bar{\gamma }}_{i}^{\prime }=1/{\bar{\gamma }}_{i}^{0}+B_{i}^{j,k,l}$. Note that the indices *j*, *k*, and *l* are omitted in ${\bar{\gamma }}_{i}^{\prime }$ for convenience. In (21), the quadruple summation is defined as (A27).

From (19), the index of the $N_{\mathrm{th}}$ best relay is selected, and the corresponding selected and combined link SNRs are defined as

(22) $$\begin{array}{cc} \gamma_{\mathrm{id}}^{N_{\max}} & =\frac{\gamma_{N_{\max}}\beta_{N_{\max}}}{\beta_{N_{\max}}+1}\\ \gamma_{\mathrm{cb}} & =\gamma_{0}+\gamma_{\mathrm{id}}^{N_{\max}} \end{array}$$

and the joint PDF of $\left\{\gamma_{N_{\max}},\beta_{N_{\max}}\right\}$ can be derived from (21) as

(23) $$\begin{array}{cc} f_{\gamma_{N_{\max}},\beta_{N_{\max}}}\left(x,y\right)\; & =\underset{i,j,k,l}{\underset{︸}{\sum \cdots \sum }}\frac{{\bar{\gamma }}_{i}^{\prime }{\left(-1\right)}^{k}}{{\bar{\gamma }}_{i}^{0}}\frac{1}{{\bar{\gamma }}_{i}^{\prime \prime }}e^{-x/{\bar{\gamma }}_{i}^{\prime \prime }}\frac{1}{{\bar{\beta }}_{i}}e^{-y/{\bar{\beta }}_{i}}u\left(x\right)u\left(y\right) \end{array}$$

with $1/{\bar{\gamma }}_{i}^{\prime }=1/{\bar{\gamma }}_{i}^{0}+B_{i}^{j,k,l}$ and ${\bar{\gamma }}_{i}^{\prime \prime }=\frac{{\bar{\gamma }}_{i}}{{\bar{\gamma }}_{i}^{0}}{\bar{\gamma }}_{i}^{\prime }$. Note that, for simplicity, the indices *j*, *k*, and *l* are omitted in ${\bar{\gamma }}_{i}^{\prime }$ and ${\bar{\gamma }}_{i}^{\prime \prime }$. Furthermore, (21) and (23) correspond to the random variable pairs ${\left\{\gamma_{i}^{0},\beta_{i}\right\}}_{i=1}^{R}$ and ${\left\{\gamma_{i},\beta_{i}\right\}}_{i=1}^{R}$, respectively. To derive (23), a transformation from $\gamma_{i}^{0}$ to $\gamma_{i}$ is applied.

### 3.2. Error Rate Expressions

When the $N_{\mathrm{th}}$ best relay is selected, the average SERs for the indirect link and the combined link can be expressed, respectively, from (23) as

(24) $$\begin{array}{cc} P_{S,\mathrm{id}}\; & =\frac{1}{\pi }\int_{0}^{(M-1)\pi /M}\left[\int_{0}^{\infty }\int_{0}^{\infty }e^{-\frac{xy}{y+1}s}f_{\gamma_{N_{\max}},\beta_{N_{\max}}}\left(x,y\right)dxdy\right]d\phi \end{array}$$

and

(25) $$\begin{array}{cc} P_{S,\mathrm{cb}}\; & =\frac{1}{\pi }\int_{0}^{(M-1)\pi /M}\frac{1}{{\bar{\gamma }}_{0}s+1}\left[\int_{0}^{\infty }\int_{0}^{\infty }e^{-\frac{xy}{y+1}s}f_{\gamma_{N_{\max}},\beta_{N_{\max}}}\left(x,y\right)dxdy\right]d\phi \end{array}$$

where $s=g_{\mathrm{PSK}}/\sin^{2}\left(\phi \right)$ and $g_{\mathrm{PSK}}=\sin^{2}\left(\pi /M\right)$. Note that the above equations, derived using the relay selection algorithm in (19), involve no approximation. This represents the exact ASER expression for the selection algorithm based on P-CSIs. Furthermore, the terms containing the inner double integrals in (24) and (25) correspond to the MGF of $\gamma_{{\mathrm{id}}_{r}}$, as given in (A2).

#### 3.2.1. Exact and Upper-Bounded ASER Expressions

Based on (23) and (A2), the average SERs in (24) and (25) can be expressed as

(26) $$\begin{array}{cc} P_{S,\mathrm{id}}\; & =\underset{i,j,k,l}{\underset{︸}{\sum \cdots \sum }}\frac{{\bar{\gamma }}_{i}^{\prime }{\left(-1\right)}^{k}}{{\bar{\gamma }}_{i}^{0}}\frac{1}{\pi }\int_{0}^{(M-1)\pi /M}M_{\mathrm{id}}\left(s|{\bar{\gamma }}_{i}^{\prime \prime },{\bar{\beta }}_{i}\right)d\phi \\ P_{S,\mathrm{cb}}\; & =\underset{i,j,k,l}{\underset{︸}{\sum \cdots \sum }}\frac{{\bar{\gamma }}_{i}^{\prime }{\left(-1\right)}^{k}}{{\bar{\gamma }}_{i}^{0}}\frac{1}{\pi }\int_{0}^{(M-1)\pi /M}\frac{1}{{\bar{\gamma }}_{0}s+1}M_{\mathrm{id}}\left(s|{\bar{\gamma }}_{i}^{\prime \prime },{\bar{\beta }}_{i}\right)d\phi . \end{array}$$

Moreover, based on (A3), the average SERs in (26) can be expressed as upper bounds of

(27) $$\begin{array}{cc} P_{S,\mathrm{id}} & <P_{S,\mathrm{id}}^{\mathrm{Up}}=\underset{i,j,k,l}{\underset{︸}{\sum \cdots \sum }}\frac{{\bar{\gamma }}_{i}^{\prime }{\left(-1\right)}^{k}}{{\bar{\gamma }}_{i}^{0}}\frac{1}{\pi }\int_{0}^{(M-1)\pi /M}M_{\mathrm{id}}^{\mathrm{Up}}\left(s|{\bar{\gamma }}_{i}^{\prime \prime },{\bar{\beta }}_{i}\right)d\phi \\ P_{S,\mathrm{cb}} & <P_{S,\mathrm{cb}}^{\mathrm{Up}}=\underset{i,j,k,l}{\underset{︸}{\sum \cdots \sum }}\frac{{\bar{\gamma }}_{i}^{\prime }{\left(-1\right)}^{k}}{{\bar{\gamma }}_{i}^{0}}\frac{1}{\pi }\int_{0}^{(M-1)\pi /M}\frac{1}{{\bar{\gamma }}_{0}s+1}M_{\mathrm{id}}^{\mathrm{Up}}\left(s|{\bar{\gamma }}_{i}^{\prime \prime },{\bar{\beta }}_{i}\right)d\phi . \end{array}$$

#### 3.2.2. Asymptotic BER Expressions

From (A7), $P_{S,\mathrm{id}}^{\mathrm{Up}}$ and $P_{S,\mathrm{cb}}^{\mathrm{Up}}$ in (27) can be asymptotically expressed for BPSK modulation as [36]

(28) $$\begin{array}{cc} P_{B,\mathrm{id}}\leq P_{B,\mathrm{id}}^{\mathrm{Asym}}\; & =\underset{i,j,k,l}{\underset{︸}{\sum \cdots \sum }}\frac{{\bar{\gamma }}_{i}^{\prime }{\left(-1\right)}^{k}}{{\bar{\gamma }}_{i}^{0}}{\left.M_{\mathrm{id}}^{\mathrm{Up}}\left(s|{\bar{\gamma }}_{i}^{\prime \prime },{\bar{\beta }}_{i}\right)\right|}_{s=4}\\ \; & =\underset{i,j,k,l}{\underset{︸}{\sum \cdots \sum }}\frac{{\bar{\gamma }}_{i}^{\prime }{\left(-1\right)}^{k}}{{\bar{\gamma }}_{i}^{0}}P_{B,\mathrm{id}}^{\mathrm{Asym}}\left({\bar{\gamma }}_{i}^{\prime \prime },{\bar{\beta }}_{i}\right) \end{array}$$

and

(29) $$\begin{array}{cc} P_{B,\mathrm{cb}}\leq P_{B,\mathrm{cb}}^{\mathrm{Asym}}\; & =\underset{i,j,k,l}{\underset{︸}{\sum \cdots \sum }}\frac{{\bar{\gamma }}_{i}^{\prime }{\left(-1\right)}^{k}}{{\bar{\gamma }}_{i}^{0}}\frac{3}{4{\bar{\gamma }}_{0}+1}P_{B,\mathrm{id}}^{\mathrm{Asym}}\left({\bar{\gamma }}_{i}^{\prime \prime },{\bar{\beta }}_{i}\right)=\frac{3}{4{\bar{\gamma }}_{0}+1}P_{B,\mathrm{id}}^{\mathrm{Asym}} \end{array}$$

where $P_{B,\mathrm{id}}^{\mathrm{Asym}}\left({\bar{\gamma }}_{i}^{\prime \prime },{\bar{\beta }}_{i}\right)={\left.M_{\mathrm{id}}^{\mathrm{Up}}\left(s|{\bar{\gamma }}_{i}^{\prime \prime },{\bar{\beta }}_{i}\right)\right|}_{s=4}$.

### 3.3. Average Link SNR Derivation

Related to (8), (10), and (22), the average link SNRs can be obtained from (23).

#### 3.3.1. SR Link

From (23), the average SR link SNR for the $N_{\mathrm{th}}$ best selected relay can be expressed as

(30) $$\begin{array}{cc} {\bar{\gamma }}_{N_{\max}}={\bar{\gamma }}_{\mathrm{sr}}^{N_{\max}}=E\left[\gamma_{N_{\max}}\right]\; & =\int_{0}^{\infty }\int_{0}^{\infty }\left(x\right)f_{\gamma_{N_{\max}},\beta_{N_{\max}}}\left(x,y\right)dxdy=\underset{i,j,k,l}{\underset{︸}{\sum \ldots \sum }}\frac{{\bar{\gamma }}_{i}^{\prime }{\left(-1\right)}^{k}{\bar{\gamma }}_{i}^{\prime \prime }}{{\bar{\gamma }}_{i}^{0}}. \end{array}$$

#### 3.3.2. RD Link

Based on (23), ${\bar{\beta }}_{N_{\max}}$ corresponding to the $N_{\mathrm{th}}$ best selected relay is obtained as

(31) $$\begin{array}{cc} {\bar{\beta }}_{N_{\max}}=E\left[\beta_{N_{\max}}\right]\; & =\int_{0}^{\infty }\int_{0}^{\infty }\left(y\right)f_{\gamma_{N_{\max}},\beta_{N_{\max}}}\left(x,y\right)dxdy=\underset{i,j,k,l}{\underset{︸}{\sum \ldots \sum }}\frac{{\bar{\gamma }}_{i}^{\prime }{\left(-1\right)}^{k}{\bar{\beta }}_{i}}{{\bar{\gamma }}_{i}^{0}}. \end{array}$$

From (23), the RD link average SNR for the $N_{\mathrm{th}}$ best selected relay can be expressed as

(32) $$\begin{array}{cc} {\bar{\gamma }}_{\mathrm{rd}}^{N_{\max}}=E\left[{\left\{\gamma_{{\mathrm{rd}}_{r}}\right\}}_{N_{\max}}\right]\; & =\sum_{i=1}^{R}\int_{0}^{\infty }\int_{0}^{\infty }\left(y\right)p_{\gamma_{i}^{0}}\left(x\right)f_{\gamma_{i}^{0}}\left(x\right)f_{\gamma_{{\mathrm{rd}}_{i}}}\left(y\right)dxdy=\underset{i,j,k,l}{\underset{︸}{\sum \ldots \sum }}\frac{{\bar{\gamma }}_{i}^{\prime }{\left(-1\right)}^{k}}{{\bar{\gamma }}_{i}^{0}}{\bar{\gamma }}_{{\mathrm{rd}}_{i}}. \end{array}$$

#### 3.3.3. Indirect Link

Based on (23), the average SNR of the indirect link corresponding to the $N_{\mathrm{th}}$ best selected relay can be expressed as [36]

(33) $$\begin{array}{cc} {\bar{\gamma }}_{\mathrm{id}}^{N_{\max}}=E\left[\gamma_{\mathrm{id}}^{N_{\max}}\right]=E\left[\frac{\gamma_{N_{\max}}\beta_{N_{\max}}}{\beta_{N_{\max}}+1}\right]\; & =\underset{i,j,k,l}{\underset{︸}{\sum \ldots \sum }}\frac{{\bar{\gamma }}_{i}^{\prime }{\left(-1\right)}^{k}}{{\bar{\gamma }}_{i}^{0}}E\left[\frac{\gamma_{i}^{\prime \prime }\beta_{i}}{\beta_{i}+1}\right] \end{array}$$

where

$$\begin{array}{c} E\left[\frac{\gamma_{i}^{\prime \prime }\beta_{i}}{\beta_{i}+1}\right]={\bar{\gamma }}_{i}^{\prime \prime }-{\bar{\gamma }}_{i}^{\prime \prime }\frac{1}{{\bar{\beta }}_{i}}\exp\left(\frac{1}{{\bar{\beta }}_{i}}\right)E_{1}\left(\frac{1}{{\bar{\beta }}_{i}}\right) \end{array}$$

and the exponential integral function is defined as $E_{1}\left(\frac{1}{\tau }\right)=\int_{1/\tau }^{\infty }\frac{e^{-y}}{y}dy$ [37,38].

### 3.4. Approximation SWIPT OAF Relay to General OAF Relay

From the analytical approach in [36], the SWIPT OAF relay system can be approximated to a conventional OAF relay system through the following steps:

- Relay selection based on partial CSIs of SR links.
- Approximation of the SWIPT indirect link as a conventional AF indirect link.
- Minimum-based approximation for the indirect link SNR pair.

Therefore, $\gamma_{{\mathrm{id}}_{r}}$ in (8) can be approximated as in [36]:

(34) $$\begin{array}{c} \gamma_{{\mathrm{id}}_{r}}=\frac{\gamma_{r}\beta_{r}}{\beta_{r}+1}\approx \frac{\gamma_{r}\gamma_{r}^{\mathrm{rd}}}{\gamma_{r}+\gamma_{r}^{\mathrm{rd}}}≃\min\left\{\gamma_{r},\gamma_{r}^{\mathrm{rd}}\right\}=\gamma_{r}^{\mathrm{OAF}} \end{array}$$

where the random variable $\gamma_{r}^{\mathrm{rd}}$ denotes the modified RD link SNR, whose PDF is given by $f_{\gamma_{r}^{\mathrm{rd}}}\left(x\right)=\frac{1}{{\bar{\gamma }}_{r}^{\mathrm{rd}}}\exp\left(-\frac{x}{{\bar{\gamma }}_{r}^{\mathrm{rd}}}\right)u\left(x\right)$. Here, ${\bar{\gamma }}_{r}^{\mathrm{rd}}$ is defined in (A8) and is a function of ${\bar{\gamma }}_{r}$ and ${\bar{\beta }}_{r}$.

#### 3.4.1. PDFs (SWIPT OAF → General OAF)

When approximating the SWIPT OAF relay system as a general OAF relay system, the random variable pair $\left\{\gamma_{r},\beta_{r}\right\}$ in (34) is replaced by $\left\{\gamma_{r},\gamma_{r}^{\mathrm{rd}}\right\}$. From (22), the $N_{\mathrm{th}}$ best selected indirect and combined link SNRs can be approximated as, respectively,

(35) $$\begin{array}{cc} \gamma_{\mathrm{id}}^{N_{\max}}=\frac{\gamma_{N_{\max}}\beta_{N_{\max}}}{\beta_{N_{\max}}+1}\; & \approx \frac{\gamma_{N_{\max}}\gamma_{N_{\max}}^{\mathrm{rd}}}{\gamma_{N_{\max}}+\gamma_{N_{\max}}^{\mathrm{rd}}}≃\min\left\{\gamma_{N_{\max}},\gamma_{N_{\max}}^{\mathrm{rd}}\right\}=\gamma_{\mathrm{id}}^{\mathrm{OAF}}\\ \gamma_{\mathrm{cb}}^{N_{\max}}=\gamma_{0}+\gamma_{\mathrm{id}}^{N_{\max}}\; & \approx \gamma_{0}+\gamma_{\mathrm{id}}^{\mathrm{OAF}}=\gamma_{\mathrm{cb}}^{\mathrm{OAF}}. \end{array}$$

Then, the joint PDF $f_{\gamma_{N_{\max}},\beta_{N_{\max}}}\left(x,y\right)$ in (23) is also modified as

(36) $$\begin{array}{cc} f_{\gamma_{N_{\max}},\gamma_{N_{\max}}^{\mathrm{rd}}}\left(x,y\right)\; & =\underset{i,j,k,l}{\underset{︸}{\sum \cdots \sum }}\frac{{\bar{\gamma }}_{i}^{\prime }{\left(-1\right)}^{k}}{{\bar{\gamma }}_{i}^{0}}\frac{1}{{\bar{\gamma }}_{i}^{\prime \prime }}e^{-x/{\bar{\gamma }}_{i}^{\prime \prime }}\frac{1}{{\bar{\gamma }}_{i}^{\mathrm{rd}}}e^{-y/{\bar{\gamma }}_{i}^{\mathrm{rd}}}u\left(x\right)u\left(y\right) \end{array}$$

where ${\bar{\gamma }}_{i}^{\mathrm{rd}}$ is a function of ${\bar{\gamma }}_{i}^{\prime \prime }$ and ${\bar{\beta }}_{i}$ within (23). Note that ${\bar{\gamma }}_{i}^{\mathrm{rd}}$ can be obtained from (A8) by substituting ${\bar{\gamma }}_{r}$ with ${\bar{\gamma }}_{i}^{\prime \prime }$ and ${\bar{\beta }}_{r}$ with ${\bar{\beta }}_{i}$.

By applying the min-approximation for the AF link pair, the PDF of $\gamma_{\mathrm{id}}^{\mathrm{OAF}}$ in (35) can be obtained from the joint PDF in (36) as

(37) $$\begin{array}{cc} f_{\gamma_{\mathrm{id}}^{\mathrm{OAF}}}\left(x\right)\; & =\underset{i,j,k,l}{\underset{︸}{\sum \cdots \sum }}\frac{{\bar{\gamma }}_{i}^{\prime }{\left(-1\right)}^{k}}{{\bar{\gamma }}_{i}^{0}}\frac{1}{{\bar{\gamma }}_{i}^{\min}}e^{-x/{\bar{\gamma }}_{i}^{\min}}u\left(x\right) \end{array}$$

where ${\bar{\gamma }}_{i}^{\min}={\left(1/{\bar{\gamma }}_{i}^{\prime \prime }+1/{\bar{\gamma }}_{i}^{\mathrm{rd}}\right)}^{-1}$. Note that, in both ${\bar{\gamma }}_{i}^{\prime \prime }$ and ${\bar{\gamma }}_{i}^{\min}$, the indices *j*, *k*, and *l* are omitted for simplicity.

By using the PDFs of $\gamma_{0}$ and $\gamma_{\mathrm{id}}^{\mathrm{OAF}}$ in (37) and performing algebraic manipulations, the PDF of $\gamma_{\mathrm{cb}}^{\mathrm{OAF}}$ in (35) can be expressed as

(38) $$\begin{array}{cc} f_{\gamma_{\mathrm{cb}}^{\mathrm{OAF}}}\left(x\right)\; & =\underset{i,j,k,l}{\underset{︸}{\sum \cdots \sum }}\frac{{\bar{\gamma }}_{i}^{\prime }{\left(-1\right)}^{k}}{{\bar{\gamma }}_{i}^{0}}\left[\frac{{\bar{\gamma }}_{0}}{{\bar{\gamma }}_{0}-{\bar{\gamma }}_{i}^{\min}}\frac{e^{-x/{\bar{\gamma }}_{0}}}{{\bar{\gamma }}_{0}}+\frac{{\bar{\gamma }}_{i}^{\min}}{{\bar{\gamma }}_{i}^{\min}-{\bar{\gamma }}_{0}}\frac{e^{-x/{\bar{\gamma }}_{i}^{\min}}}{{\bar{\gamma }}_{i}^{\min}}\right]u\left(x\right). \end{array}$$

#### 3.4.2. ABER Expressions Based on Min-Approximation of OAF Links

Using (37) and (38), the approximated average BERs for the indirect and combined links are given by

(39) $$\begin{array}{cc} P_{B,\mathrm{id}}^{\mathrm{OAF}}\; & =\alpha \int_{0}^{\infty }Q\left(\sqrt{\beta \gamma }\right)f_{\gamma_{\mathrm{id}}^{\mathrm{OAF}}}\left(\gamma \right)d\gamma =\underset{i,j,k,l}{\underset{︸}{\sum \cdots \sum }}\frac{{\bar{\gamma }}_{i}^{\prime }{\left(-1\right)}^{k}}{{\bar{\gamma }}_{i}^{0}}\frac{\alpha }{2}\left(1-\sqrt{\frac{\beta {\bar{\gamma }}_{i}^{\min}/2}{\beta {\bar{\gamma }}_{i}^{\min}/2+1}}\right) \end{array}$$

and

(40) $$\begin{array}{cc} P_{B,\mathrm{cb}}^{\mathrm{OAF}}\; & =\alpha \int_{0}^{\infty }Q\left(\sqrt{\beta \gamma }\right)f_{\gamma_{\mathrm{cb}}^{\mathrm{OAF}}}\left(\gamma \right)d\gamma \\ \; & =\underset{i,j,k,l}{\underset{︸}{\sum \cdots \sum }}\frac{{\bar{\gamma }}_{i}^{\prime }{\left(-1\right)}^{k}}{{\bar{\gamma }}_{i}^{0}}\frac{{\bar{\gamma }}_{0}}{{\bar{\gamma }}_{0}-{\bar{\gamma }}_{i}^{\min}}\frac{\alpha }{2}\left(1-\sqrt{\frac{\beta {\bar{\gamma }}_{0}/2}{\beta {\bar{\gamma }}_{0}/2+1}}\right)\\ \; & +\underset{i,j,k,l}{\underset{︸}{\sum \cdots \sum }}\frac{{\bar{\gamma }}_{i}^{\prime }{\left(-1\right)}^{k}}{{\bar{\gamma }}_{i}^{0}}\frac{{\bar{\gamma }}_{i}^{\min}}{{\bar{\gamma }}_{i}^{\min}-{\bar{\gamma }}_{0}}\frac{\alpha }{2}\left(1-\sqrt{\frac{\beta {\bar{\gamma }}_{i}^{\min}/2}{\beta {\bar{\gamma }}_{i}^{\min}/2+1}}\right). \end{array}$$

where α=1 and β=2 for BPSK.

## 4. Performance Analysis for the SWIPT OAF Relaying System: P-CSI Based on RD Link

Note that although this paper primarily focuses on the downlink relay selection scenario (S→R→D), the same relay selection strategy can be straightforwardly applied to the uplink configuration (D→R→S), since the underlying structural model and signal expressions remain symmetric.

In this section, we investigate the relay selection strategy for SWIPT OAF relaying systems based on P-CSI of the RD link channels and analyze the associated error rate performance. Following the block structure illustrated in Figure 2, we describe the principle of selecting the optimal relay based solely on RD link information. To this end, we define a ‘D→R subphase’, which is analogous to the ‘S→R subphase’ depicted in Figure 2. During this subphase, the received signal at the *r*th relay is modeled as

(41) $$\begin{array}{cc} y_{{\mathrm{dr}}_{r}} & \;=h_{R+r}x_{d}+n_{{\mathrm{dr}}_{r}}, \end{array}$$

where $x_{d}$ is a known reference signal transmitted by the destination node, satisfying $E\left[x_{d}\right]=0$ and $E\left[{\left|x_{d}\right|}^{2}\right]=P_{d}$. The term $n_{{\mathrm{dr}}_{r}}$ denotes the additive white Gaussian noise (AWGN) at the *r*th relay, with $E\left[n_{{\mathrm{dr}}_{r}}\right]=0$ and $E\left[{\left|n_{{\mathrm{dr}}_{r}}\right|}^{2}\right]=\sigma^{2}$. Also, from ‘D→R→D subphase’, the received signal at the destination can be expressed as

(42) $$\begin{array}{cc} y_{{\mathrm{drd}}_{r}}\; & =h_{R+r}x_{{\mathrm{rd}}_{r}}+n_{{\mathrm{rd}}_{r}}=h_{R+r}\kappa_{{\mathrm{rd}}_{r}}\sqrt{1-\rho_{0}}x_{r}^{\mathrm{RID}}+n_{{\mathrm{rd}}_{r}} \end{array}$$

where $x_{{\mathrm{rd}}_{r}}=\kappa_{{\mathrm{rd}}_{r}}\sqrt{1-\rho_{0}}x_{r}^{\mathrm{RID}}$, $\kappa_{{\mathrm{rd}}_{r}}=\sqrt{\frac{\eta \rho_{0}P_{d}{\left|h_{R+r}\right|}^{2}}{1-\rho_{0}}}$, and $n_{{\mathrm{rd}}_{r}}$ represents the AWGN at the destination node with $E\left[n_{{\mathrm{rd}}_{r}}\right]=0$ and $E\left[{\left|n_{{\mathrm{rd}}_{r}}\right|}^{2}\right]=\sigma^{2}$. From (42), the received SNR and the perfectly estimated channel gain can be expressed as

(43) $$\begin{array}{cc} {\hat{\gamma }}_{{\mathrm{drd}}_{r}} & =\frac{\eta \rho_{0}P_{d}{\left|h_{R+r}\right|}^{4}}{\sigma^{2}}\\ {\hat{h}}_{{\mathrm{drd}}_{r}} & =\sqrt{\eta \rho_{0}P_{d}}{\left|h_{R+r}\right|}^{2}. \end{array}$$

From (11), (42), and (43), it follows that

(44) $$\begin{array}{cc} \gamma_{{\mathrm{rd}}_{r}}^{0} & =\frac{\eta \rho_{0}P_{s}{\left|h_{R+r}\right|}^{2}}{\sigma^{2}}\propto \beta_{r}^{0}\propto \sqrt{{\hat{\gamma }}_{{\mathrm{drd}}_{r}}}\propto {\hat{h}}_{{\mathrm{drd}}_{r}}\propto {\left|h_{R+r}\right|}^{2}. \end{array}$$

The relay selection is performed at the destination during the setup process of dual-hop multiple SWIPT relay systems. Accordingly, the destination node selects the best relay from ${\left\{{\hat{h}}_{{\mathrm{drd}}_{r}}\right\}}_{r=1}^{R}$. The relay selection algorithm is given by

(45) $$\begin{array}{cc} i\; & =\arg\max_{r\in \{1,2,\cdots ,R\}}\left\{{\hat{h}}_{{\mathrm{drd}}_{r}}\right\}=\arg\max_{r\in \{1,2,\cdots ,R\}}\left\{{\left|h_{R+r}\right|}^{2}\right\}=\arg\max_{r\in \{1,2,\cdots ,R\}}\left\{\beta_{r}^{0}\right\}. \end{array}$$

### 4.1. Nth Best Selection Probability

To derive the exact ASER expression for the SWIPT OAF relay systems, we first consider the selection algorithm based on P-CSIs using RD link SNRs. Using ${\left\{\beta_{r}^{0}\right\}}_{r=1}^{R}$ from (11), the relay selection algorithm can be expressed as

(46) $$\begin{array}{cc} i & \;=\arg\left(N_{\mathrm{th}}\max{\left\{\beta_{r}^{0}\right\}}_{r=1}^{R}\right). \end{array}$$

Note that in (46), the destination node performs the relay selection process using ${\left\{\beta_{r}^{0}\right\}}_{r=1}^{R}$ prior to the PS optimization. In this paper, we assume that all relay nodes initialize the PS factor as ${\left.\rho_{r}\right|}_{r=1}^{R}=\rho_{0}$. After the relay selection in (46), each relay node performs PS optimization independently. Therefore, the random variable pair $\left\{\gamma_{i},\beta_{i}\right\}$ with the optimized $\rho_{i}$ is used during data transmission.

#### 4.1.1. Relay Selection Probability for the *i*th Relay

The selection problem in (46) corresponds to choosing the $N_{\mathrm{th}}$ largest value among *R* independent random variables. Each of the *R* relays can be selected as the $N_{\mathrm{th}}$ best relay. Using (A23) and (A24), the selection probability of the random variable $\beta_{i}^{0}$ can be expressed as

(47) pβi0x=∑j=1R−1N−1∑k=0R−N(−1)k∑l=1R−Nkexp−xBij,k,l

where

$$B_{i}^{j,k,l}=\sum_{m=1}^{N-1}1/{\bar{\beta }}_{\lambda_{i,m}^{N-1,j}}^{0}+\sum_{m=1}^{k}1/{\bar{\beta }}_{{\left(\lambda_{i}^{\bar{N-1},j}\right)}_{m}^{k,l}}^{0},$$

with the convention that ${\left.\sum_{m=1}^{k}\left(\cdot \right)\right|}_{k=0}=0$, and ${\left.{\bar{\beta }}_{r}^{0}\right|}_{r=1}^{R}$ is given in (10) with ${\left.\rho_{r}\right|}_{r=1}^{R}=\rho_{0}$. The detailed derivation of (47) is provided in Appendix B.

#### 4.1.2. Joint PDF

From (46) and (47), the joint PDF of selecting the $N_{\mathrm{th}}$ random variable pair from ${\left\{\gamma_{i},\beta_{i}^{0}\right\}}_{i=1}^{R}$ can be expressed as

(48) $$\begin{array}{cc} f_{\gamma_{N_{\max}},\beta_{N_{\max}}^{0}}\left(x,y\right)\; & =\sum_{i=1}^{R}p_{\beta_{i}^{0}}\left(y\right)f_{\gamma_{i}}\left(x\right)f_{\beta_{i}^{0}}\left(y\right)\\ \; & =\underset{i,j,k,l}{\underset{︸}{\sum \cdots \sum }}\frac{{\bar{\beta }}_{i}^{\prime }{\left(-1\right)}^{k}}{{\bar{\beta }}_{i}^{0}}\frac{1}{{\bar{\gamma }}_{i}}e^{-x/{\bar{\gamma }}_{i}}\frac{1}{{\bar{\beta }}_{i}^{\prime }}e^{-y/{\bar{\beta }}_{i}^{\prime }}u\left(x\right)u\left(y\right) \end{array}$$

with $1/{\bar{\beta }}_{i}^{\prime }=1/{\bar{\beta }}_{i}^{0}+B_{i}^{j,k,l}$. For convenience, the indices *j*, *k*, and *l* in ${\bar{\beta }}_{i}^{\prime }$ are omitted.

From (46), the $N_{\mathrm{th}}$ best relay index is selected, and the corresponding selected link SNR can be defined as $\gamma_{\mathrm{id}}^{N_{\max}}=\frac{\gamma_{N_{\max}}\beta_{N_{\max}}}{\beta_{N_{\max}}+1}$. Then, the joint PDF of $\left\{\gamma_{N_{\max}},\beta_{N_{\max}}\right\}$ can be derived from (48) as

(49) $$\begin{array}{cc} f_{\gamma_{N_{\max}},\beta_{N_{\max}}}\left(x,y\right)\; & =\underset{i,j,k,l}{\underset{︸}{\sum \cdots \sum }}\frac{{\bar{\beta }}_{i}^{\prime }{\left(-1\right)}^{k}}{{\bar{\beta }}_{i}^{0}}\frac{1}{{\bar{\gamma }}_{i}}e^{-x/{\bar{\gamma }}_{i}}\frac{1}{{\bar{\beta }}_{i}^{\prime \prime }}e^{-y/{\bar{\beta }}_{i}^{\prime \prime }}u\left(x\right)u\left(y\right) \end{array}$$

where $1/{\bar{\beta }}_{i}^{\prime }=1/{\bar{\beta }}_{i}^{0}+B_{i}^{j,k,l}$ and ${\bar{\beta }}_{i}^{\prime \prime }=\frac{{\bar{\beta }}_{i}}{{\bar{\beta }}_{i}^{0}}{\bar{\beta }}_{i}^{\prime }$. Note that, in ${\bar{\beta }}_{i}^{\prime }$ and ${\bar{\beta }}_{i}^{\prime \prime }$, the indices *j*, *k*, and *l* are omitted for convenience. To derive (49) from (48), a transformation from $\beta_{i}^{0}$ to $\beta_{i}$ is applied.

### 4.2. Error Rate Expressions

When the $N_{\mathrm{th}}$ best relay is selected, the average SERs for the indirect and combined links can be expressed as

(50) $$\begin{array}{cc} P_{S,\mathrm{id}}\; & =\frac{1}{\pi }\int_{0}^{(M-1)\pi /M}\left[\int_{0}^{\infty }\int_{0}^{\infty }e^{-\frac{xy}{y+1}s}f_{\gamma_{N_{\max}},\beta_{N_{\max}}}\left(x,y\right)dxdy\right]d\phi \end{array}$$

and

(51) $$\begin{array}{cc} P_{S,\mathrm{cb}}\; & =\frac{1}{\pi }\int_{0}^{(M-1)\pi /M}\frac{1}{{\bar{\gamma }}_{0}s+1}\left[\int_{0}^{\infty }\int_{0}^{\infty }e^{-\frac{xy}{y+1}s}f_{\gamma_{N_{\max}},\beta_{N_{\max}}}\left(x,y\right)dxdy\right]d\phi . \end{array}$$

#### 4.2.1. Exact and Upper Bounded ASER Expressions

From (49) and (A2), the average SERs in (50) and (51) can be expressed as follows:

(52) $$\begin{array}{cc} P_{S,\mathrm{id}}\; & =\underset{i,j,k,l}{\underset{︸}{\sum \cdots \sum }}\frac{{\bar{\beta }}_{i}^{\prime }{\left(-1\right)}^{k}}{{\bar{\beta }}_{i}^{0}}\frac{1}{\pi }\int_{0}^{(M-1)\pi /M}M_{\mathrm{id}}\left(s|{\bar{\gamma }}_{i},{\bar{\beta }}_{i}^{\prime \prime }\right)d\phi \\ P_{S,\mathrm{cb}}\; & =\underset{i,j,k,l}{\underset{︸}{\sum \cdots \sum }}\frac{{\bar{\beta }}_{i}^{\prime }{\left(-1\right)}^{k}}{{\bar{\beta }}_{i}^{0}}\frac{1}{\pi }\int_{0}^{(M-1)\pi /M}\frac{1}{{\bar{\gamma }}_{0}s+1}M_{\mathrm{id}}\left(s|{\bar{\gamma }}_{i},{\bar{\beta }}_{i}^{\prime \prime }\right)d\phi . \end{array}$$

Moreover, from (A3), the average SERs in (52) can be expressed as upper bounds:

(53) $$\begin{array}{cc} P_{S,\mathrm{id}}\; & <P_{S,\mathrm{id}}^{\mathrm{Up}}=\underset{i,j,k,l}{\underset{︸}{\sum \cdots \sum }}\frac{{\bar{\beta }}_{i}^{\prime }{\left(-1\right)}^{k}}{{\bar{\beta }}_{i}^{0}}\frac{1}{\pi }\int_{0}^{(M-1)\pi /M}M_{\mathrm{id}}^{\mathrm{Up}}\left(s|{\bar{\gamma }}_{i},{\bar{\beta }}_{i}^{\prime \prime }\right)d\phi \\ P_{S,\mathrm{cb}}\; & <P_{S,\mathrm{cb}}^{\mathrm{Up}}=\underset{i,j,k,l}{\underset{︸}{\sum \cdots \sum }}\frac{{\bar{\beta }}_{i}^{\prime }{\left(-1\right)}^{k}}{{\bar{\beta }}_{i}^{0}}\frac{1}{\pi }\int_{0}^{(M-1)\pi /M}\frac{1}{{\bar{\gamma }}_{0}s+1}M_{\mathrm{id}}^{\mathrm{Up}}\left(s|{\bar{\gamma }}_{i},{\bar{\beta }}_{i}^{\prime \prime }\right)d\phi . \end{array}$$

#### 4.2.2. Asymptotic BER Expressions

With the help of (14-4-18) in [39] and after some manipulations, $P_{S,\mathrm{id}}^{\mathrm{Up}}$ and $P_{S,\mathrm{cb}}^{\mathrm{Up}}$ in (53) can be asymptotically expressed as [36]:

(54) $$\begin{array}{cc} P_{B,\mathrm{id}}<P_{B,\mathrm{id}}^{\mathrm{Up}}⪅P_{B,\mathrm{id}}^{\mathrm{Asym}}\; & =\underset{i,j,k,l}{\underset{︸}{\sum \cdots \sum }}\frac{{\bar{\beta }}_{i}^{\prime }{\left(-1\right)}^{k}}{{\bar{\beta }}_{i}^{0}}{\left.M_{\mathrm{id}}^{\mathrm{Up}}\left(s|{\bar{\gamma }}_{i},{\bar{\beta }}_{i}^{\prime \prime }\right)\right|}_{s=4}\\ \; & =\underset{i,j,k,l}{\underset{︸}{\sum \cdots \sum }}\frac{{\bar{\beta }}_{i}^{\prime }{\left(-1\right)}^{k}}{{\bar{\beta }}_{i}^{0}}P_{B,\mathrm{id}}^{\mathrm{Asym}}\left({\bar{\gamma }}_{i},{\bar{\beta }}_{i}^{\prime \prime }\right) \end{array}$$

and

(55) $$\begin{array}{cc} P_{B,\mathrm{cb}}<P_{B,\mathrm{cb}}^{\mathrm{Up}}⪅P_{B,\mathrm{cb}}^{\mathrm{Asym}}\; & =\underset{i,j,k,l}{\underset{︸}{\sum \cdots \sum }}\frac{{\bar{\beta }}_{i}^{\prime }{\left(-1\right)}^{k}}{{\bar{\beta }}_{i}^{0}}\frac{3}{4{\bar{\gamma }}_{0}+1}P_{B,\mathrm{id}}^{\mathrm{Asym}}\left({\bar{\gamma }}_{i},{\bar{\beta }}_{i}^{\prime \prime }\right)=\frac{3}{4{\bar{\gamma }}_{0}+1}P_{B,\mathrm{id}}^{\mathrm{Asym}} \end{array}$$

where $P_{B,\mathrm{id}}^{\mathrm{Asym}}\left({\bar{\gamma }}_{i},{\bar{\beta }}_{i}^{\prime \prime }\right)={\left.M_{\mathrm{id}}^{\mathrm{Up}}\left(s|{\bar{\gamma }}_{i},{\bar{\beta }}_{i}^{\prime \prime }\right)\right|}_{s=4}$.

### 4.3. Average Link SNR Derivation

#### 4.3.1. SR Link

For the $N_{\mathrm{th}}$ best selected relay, the average SR link SNR can be expressed from (49) as

(56) $$\begin{array}{cc} {\bar{\gamma }}_{N_{\max}}=E\left[\gamma_{N_{\max}}\right]={\bar{\gamma }}_{\mathrm{sr}}^{N_{\max}}\; & =\underset{i,j,k,l}{\underset{︸}{\sum \cdots \sum }}\frac{{\bar{\beta }}_{i}^{\prime }{\left(-1\right)}^{k}{\bar{\gamma }}_{i}}{{\bar{\beta }}_{i}^{0}}. \end{array}$$

#### 4.3.2. RD Link

For the $N_{\mathrm{th}}$ best selected relay, ${\bar{\beta }}_{N_{\max}}$ can be obtained from (49) as

(57) $$\begin{array}{cc} {\bar{\beta }}_{N_{\max}}=E\left[\beta_{N_{\max}}\right]\; & =\underset{i,j,k,l}{\underset{︸}{\sum \cdots \sum }}\frac{{\bar{\beta }}_{i}^{\prime }{\left(-1\right)}^{k}{\bar{\beta }}_{i}^{\prime \prime }}{{\bar{\beta }}_{i}^{0}}. \end{array}$$

From (49), the RD link average SNR for the $N_{\mathrm{th}}$ best selected relay can be expressed as

(58) $$\begin{array}{cc} {\bar{\gamma }}_{\mathrm{rd}}^{N_{\max}}=E\left[{\left\{\gamma_{{\mathrm{rd}}_{r}}\right\}}_{N_{\max}}\right]\; & =\underset{i,j,k,l}{\underset{︸}{\sum \cdots \sum }}\frac{{\bar{\beta }}_{i}^{\prime }{\left(-1\right)}^{k}}{{\bar{\beta }}_{i}^{0}}{\bar{\gamma }}_{{\mathrm{rd}}_{i}}=\underset{i,j,k,l}{\underset{︸}{\sum \cdots \sum }}\frac{{\bar{\beta }}_{i}^{\prime }{\left(-1\right)}^{k}}{{\bar{\beta }}_{i}^{0}}\frac{{\bar{\gamma }}_{i}}{\Omega_{i}}{\bar{\beta }}_{i}^{\prime \prime }. \end{array}$$

#### 4.3.3. Indirect Link

For the $N_{\mathrm{th}}$ best selected relay, the indirect link average SNR can be expressed from (49) as

(59) $$\begin{array}{cc} {\bar{\gamma }}_{\mathrm{id}}^{N_{\max}}=E\left[\gamma_{\mathrm{id}}^{N_{\max}}\right]=E\left[\frac{\gamma_{N_{\max}}\beta_{N_{\max}}}{\beta_{N_{\max}}+1}\right]\; & =\underset{i,j,k,l}{\underset{︸}{\sum \cdots \sum }}\frac{{\bar{\beta }}_{i}^{\prime }{\left(-1\right)}^{k}}{{\bar{\beta }}_{i}^{0}}E\left[\frac{\gamma_{i}\beta_{i}^{\prime \prime }}{\beta_{i}^{\prime \prime }+1}\right] \end{array}$$

where

$$\begin{array}{c} E\left[\frac{\gamma_{i}\beta_{i}^{\prime \prime }}{\beta_{i}^{\prime \prime }+1}\right]={\bar{\gamma }}_{i}-{\bar{\gamma }}_{i}\frac{1}{{\bar{\beta }}_{i}^{\prime \prime }}\exp\left(\frac{1}{{\bar{\beta }}_{i}^{\prime \prime }}\right)E_{1}\left(\frac{1}{{\bar{\beta }}_{i}^{\prime \prime }}\right). \end{array}$$

### 4.4. Approximation SWIPT OAF Relay to General OAF Relay

From the analytical approach in [36], the SWIPT OAF relay system can be approximated as a general OAF relay system through the following steps:

- Relay selection based on partial CSI of RD links.
- Approximation of the SWIPT indirect link as a conventional AF indirect link.
- Minimum-based approximation for the indirect link SNR pair.

#### 4.4.1. PDFs (SWIPT OAF → General OAF)

Under the same assumption in Section 3.4, the joint-PDF $f_{\gamma_{N_{\max}},\beta_{N_{\max}}}\left(x,y\right)$ in (49) is also modified as follows:

(60) $$\begin{array}{cc} f_{\gamma_{N_{\max}},\gamma_{N_{\max}}^{\mathrm{rd}}}\left(x,y\right)\; & =\underset{i,j,k,l}{\underset{︸}{\sum \cdots \sum }}\frac{{\bar{\beta }}_{i}^{\prime }{\left(-1\right)}^{k}}{{\bar{\beta }}_{i}^{0}}\frac{1}{{\bar{\gamma }}_{i}}e^{-x/{\bar{\gamma }}_{i}}\frac{1}{{\bar{\gamma }}_{i}^{\mathrm{rd}}}e^{-y/{\bar{\gamma }}_{i}^{\mathrm{rd}}}u\left(x\right)u\left(y\right) \end{array}$$

with $1/{\bar{\beta }}_{i}^{\prime }=1/{\bar{\beta }}_{i}^{0}+B_{i}^{j,k,l}$ and ${\bar{\beta }}_{i}^{\prime \prime }=\frac{{\bar{\beta }}_{i}}{{\bar{\beta }}_{i}^{0}}{\bar{\beta }}_{i}^{\prime }$. Note that, for convenience, the indices *j*, *k*, and *l* in ${\bar{\beta }}_{i}^{\prime }$, ${\bar{\beta }}_{i}^{\prime \prime }$, and ${\bar{\gamma }}_{i}^{\mathrm{rd}}$ are omitted. Moreover, ${\bar{\gamma }}_{i}^{\mathrm{rd}}$ in (60) is a function of ${\bar{\gamma }}_{i}$ and ${\bar{\beta }}_{i}^{\prime \prime }$ as defined in (49). It should be noted that ${\bar{\gamma }}_{i}^{\mathrm{rd}}$ can be obtained from (A8) by replacing ${\bar{\gamma }}_{r}$ with ${\bar{\gamma }}_{i}$ and ${\bar{\beta }}_{r}$ with ${\bar{\beta }}_{i}^{\prime \prime }$.

By applying the min-approximation for the AF link pair, from the joint-PDF in (49), the PDF of $\gamma_{\mathrm{id}}^{\mathrm{OAF}}$ can be derived as

(61) $$\begin{array}{cc} f_{\gamma_{\mathrm{id}}^{\mathrm{OAF}}}\left(x\right)\; & =\underset{i,j,k,l}{\underset{︸}{\sum \cdots \sum }}\frac{{\bar{\beta }}_{i}^{\prime }{\left(-1\right)}^{k}}{{\bar{\beta }}_{i}^{0}}\frac{1}{{\bar{\gamma }}_{i}^{\min}}e^{-x/{\bar{\gamma }}_{i}^{\min}}u\left(x\right) \end{array}$$

with ${\bar{\gamma }}_{i}^{\min}={\left(1/{\bar{\gamma }}_{i}+1/{\bar{\gamma }}_{i}^{\mathrm{rd}}\right)}^{-1}$. Note that, in ${\bar{\beta }}_{i}^{\prime }$, ${\bar{\gamma }}_{i}^{\mathrm{rd}}$, and ${\bar{\gamma }}_{i}^{\min}$, the indices *j*, *k*, and *l* are omitted for convenience.

By utilizing the PDFs of $\gamma_{0}$ and $\gamma_{\mathrm{id}}^{\mathrm{OAF}}$ in (61) and applying algebraic manipulations, the PDF of $\gamma_{\mathrm{cb}}^{\mathrm{OAF}}$ can be accordingly expressed as

(62) $$\begin{array}{cc} f_{\gamma_{\mathrm{cb}}^{\mathrm{OAF}}}\left(x\right)\; & =\underset{i,j,k,l}{\underset{︸}{\sum \cdots \sum }}\frac{{\bar{\beta }}_{i}^{\prime }{\left(-1\right)}^{k}}{{\bar{\beta }}_{i}^{0}}\left[\frac{{\bar{\gamma }}_{0}}{{\bar{\gamma }}_{0}-{\bar{\gamma }}_{i}^{\min}}\frac{e^{-x/{\bar{\gamma }}_{0}}}{{\bar{\gamma }}_{0}}+\frac{{\bar{\gamma }}_{i}^{\min}}{{\bar{\gamma }}_{i}^{\min}-{\bar{\gamma }}_{0}}\frac{e^{-x/{\bar{\gamma }}_{i}^{\min}}}{{\bar{\gamma }}_{i}^{\min}}\right]u\left(x\right). \end{array}$$

#### 4.4.2. Approximated Closed-Form Expression of ABRE

From (61) and (62), the approximate average BERs for the indirect and combined links can be expressed, respectively, as

(63) $$\begin{array}{cc} P_{B,\mathrm{id}}^{\mathrm{OAF}}\; & =\alpha \int_{0}^{\infty }Q\left(\sqrt{\beta \gamma }\right)f_{\gamma_{\mathrm{id}}^{\mathrm{OAF}}}\left(\gamma \right)d\gamma =\underset{i,j,k,l}{\underset{︸}{\sum \cdots \sum }}\frac{{\bar{\beta }}_{i}^{\prime }{\left(-1\right)}^{k}}{{\bar{\beta }}_{i}^{0}}\frac{\alpha }{2}\left(1-\sqrt{\frac{\beta {\bar{\gamma }}_{i}^{\min}/2}{\beta {\bar{\gamma }}_{i}^{\min}/2+1}}\right) \end{array}$$

and

(64) $$\begin{array}{cc} P_{B,\mathrm{cb}}^{\mathrm{OAF}}\; & =\alpha \int_{0}^{\infty }Q\left(\sqrt{\beta \gamma }\right)f_{\gamma_{\mathrm{cb}}^{\mathrm{OAF}}}\left(\gamma \right)d\gamma \\ \; & =\underset{i,j,k,l}{\underset{︸}{\sum \cdots \sum }}\frac{{\bar{\beta }}_{i}^{\prime }{\left(-1\right)}^{k}}{{\bar{\beta }}_{i}^{0}}\frac{{\bar{\gamma }}_{0}}{{\bar{\gamma }}_{0}-{\bar{\gamma }}_{i}^{\min}}\frac{\alpha }{2}\left(1-\sqrt{\frac{\beta {\bar{\gamma }}_{0}/2}{\beta {\bar{\gamma }}_{0}/2+1}}\right)\\ \; & +\underset{i,j,k,l}{\underset{︸}{\sum \cdots \sum }}\frac{{\bar{\beta }}_{i}^{\prime }{\left(-1\right)}^{k}}{{\bar{\beta }}_{i}^{0}}\frac{{\bar{\gamma }}_{i}^{\min}}{{\bar{\gamma }}_{i}^{\min}-{\bar{\gamma }}_{0}}\frac{\alpha }{2}\left(1-\sqrt{\frac{\beta {\bar{\gamma }}_{i}^{\min}/2}{\beta {\bar{\gamma }}_{i}^{\min}/2+1}}\right) \end{array}$$

where α=1 and β=2 for BPSK.

Note that the derived error rate expressions have the following relationship.

$$\begin{array}{cc} P_{S,\mathrm{id}} & <P_{S,\mathrm{id}}^{\mathrm{Up}}⪅P_{B,\mathrm{id}}^{\mathrm{Asym}}\\ P_{S,\mathrm{cb}} & <P_{S,\mathrm{cb}}^{\mathrm{Up}}⪅P_{B,\mathrm{id}}^{\mathrm{Asym}}\\ P_{S,\mathrm{id}}^{\mathrm{Up}} & \approx \underset{\mathrm{High}\;\mathrm{SNR}}{\underset{︸}{P_{B,\mathrm{id}}^{\mathrm{OAF}}=P_{B,\mathrm{id}}^{\mathrm{Asym}}}}\\ P_{S,\mathrm{id}}^{\mathrm{Up}} & \approx \underset{\mathrm{High}\;\mathrm{SNR}}{\underset{︸}{P_{B,\mathrm{cb}}^{\mathrm{OAF}}=P_{B,\mathrm{cb}}^{\mathrm{Asym}}}} \end{array}$$

## 5. Numerical and Simulation Results

In this section, we numerically evaluate the analytical expressions and validate them through extensive Monte Carlo simulations. The simulations assume the multi-relay SWIPT setup described in Section 2, using MATLAB R2024a. We consider BPSK modulation (M=2), and all channels ${\left\{h_{r}\right\}}_{r=0}^{2R}$ are independently Rayleigh-faded with average powers listed in Table 1. The channel and noise realizations, including ${\left\{n_{0},n_{r},n_{R+r},n_{c_{r}}\right\}}_{r=1}^{R}$, are independently generated $10^{8}$ times, assuming AWGN with variance $\sigma^{2}$ at all receivers. The average SNR is defined as $\mathrm{SNR}={\bar{\gamma }}_{0}$. In the plots, the blue and red BER curves represent the results for the indirect and combined links, respectively, as derived numerically. Simulation curves labeled as ‘Simulation’ are based on exact application of the amplification factor $\kappa_{r}$ in (5). Two distinct channel environments are tested: ‘Ch. Model$\;=\left[1,1,X\right]$’ where RD channels vary with *R*, and ‘Ch. Model$\;=\left[1,X,1\right]$’ where SR channels vary.

In addition, the energy conversion efficiency η is set at 1.0 [1,36]. It is worth noting that various prior works have adopted different values for the energy conversion efficiency η, such as 0.2 in [31], 0.5 in [2], 0.7 in [11], and even 0.5 to 0.8 in [40], while some works have considered the ideal case with η=1.0 as in [1,36]. In our simulation setup, we intentionally adopt η=1.0 to represent the best-case scenario. This assumption allows for the evaluation of the theoretical upper bound on system performance, which can be used as a benchmark for comparison with more practical configurations. The results presented in this section thus reflect the maximum achievable performance under ideal energy harvesting conditions, assuming future advancements in EH technology. A discussion on how lower efficiency values may affect system behavior is provided in the response letter and will be explored in future work.

### 5.1. SWIPT OAF Relaying System Using P-CSIs Based on SR Links

**Figure 3:**
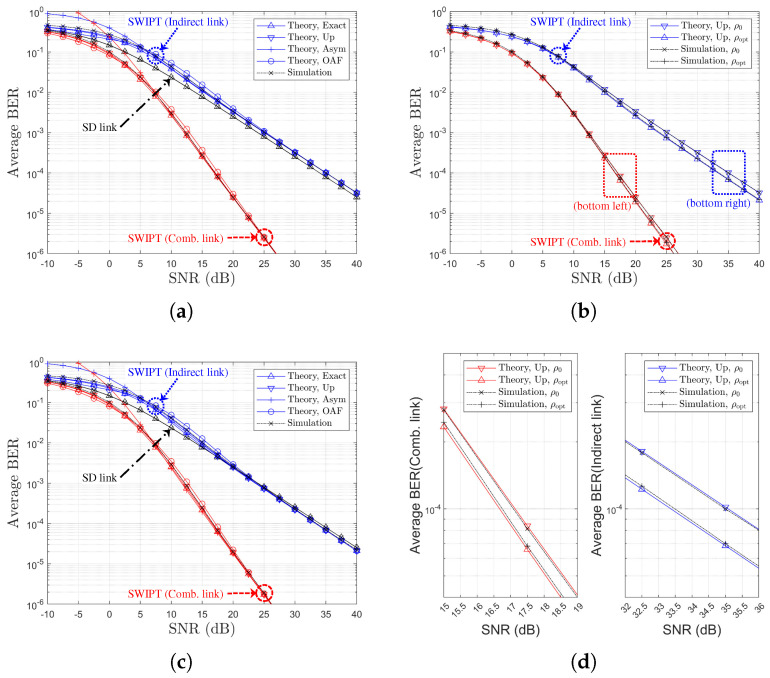
BER Performance comparison with respect to SNRs and PS optimization (SR-link-based relay selection, η=1.0, $\rho_{0}=0.5$, R=4, $N_{\mathrm{th}}=1$, Ch. Model$\;=\left[1,1,X\right]$): (**a**) $\rho_{r}=\rho_{0}$ (both links), (**b**) $\rho_{r}=\rho_{0}$ vs. $\rho_{r}=\rho_{{\mathrm{opt}}_{r}}$ (both links), (**c**) $\rho_{r}=\rho_{{\mathrm{opt}}_{r}}$ (both links), and (**d**) $\rho_{r}=\rho_{0}$ vs. $\rho_{r}=\rho_{{\mathrm{opt}}_{r}}$ (separate link).

**Figure 4:**
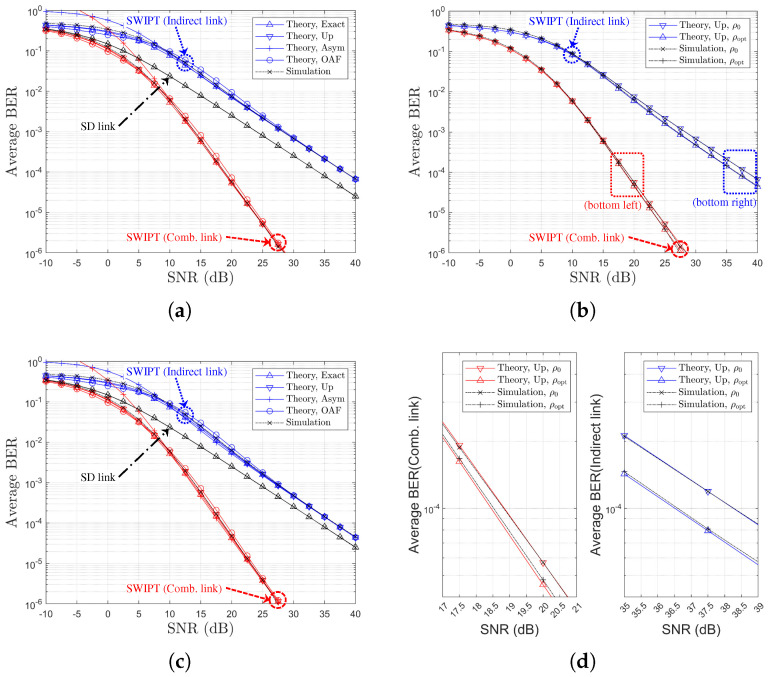
BER Performance comparison with respect to SNRs and PS optimization (SR-link-based relay selection, η=1.0, $\rho_{0}=0.5$, R=4, $N_{\mathrm{th}}=2$, Ch. Model$\;=\left[1,1,X\right]$): (**a**) $\rho_{r}=\rho_{0}$ (both links), (**b**) $\rho_{r}=\rho_{0}$ vs. $\rho_{r}=\rho_{{\mathrm{opt}}_{r}}$ (both links), (**c**) $\rho_{r}=\rho_{{\mathrm{opt}}_{r}}$ (both links), and (**d**) $\rho_{r}=\rho_{0}$ vs. $\rho_{r}=\rho_{{\mathrm{opt}}_{r}}$ (separate link).

**Figure 5:**
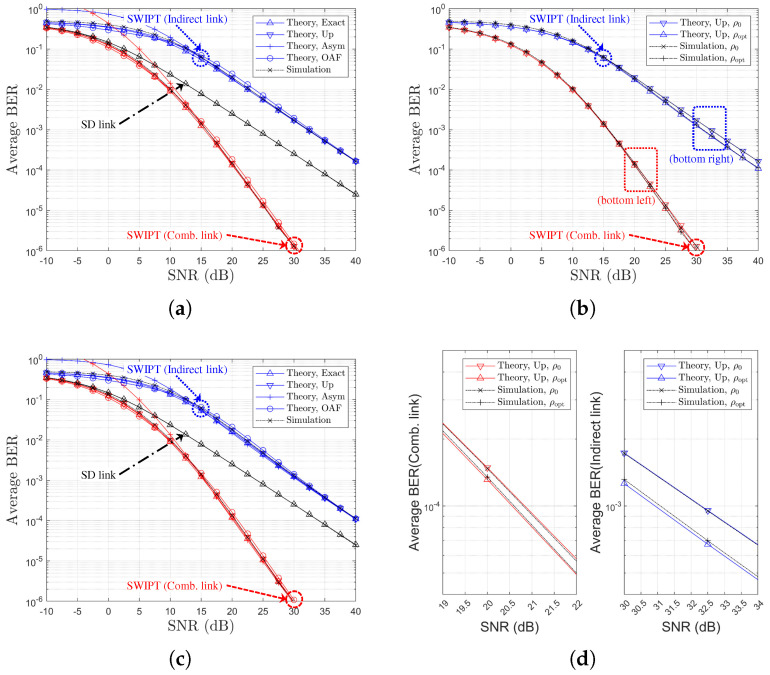
BER Performance comparison with respect to SNRs and PS optimization (SR-link-based relay selection, η=1.0, $\rho_{0}=0.5$, R=4, $N_{\mathrm{th}}=3$, Ch. Model$\;=\left[1,1,X\right]$): (**a**) $\rho_{r}=\rho_{0}$ (both links), (**b**) $\rho_{r}=\rho_{0}$ vs. $\rho_{r}=\rho_{{\mathrm{opt}}_{r}}$ (both links), (**c**) $\rho_{r}=\rho_{{\mathrm{opt}}_{r}}$ (both links), and (**d**) $\rho_{r}=\rho_{0}$ vs. $\rho_{r}=\rho_{{\mathrm{opt}}_{r}}$ (separate link).

**Figure 6:**
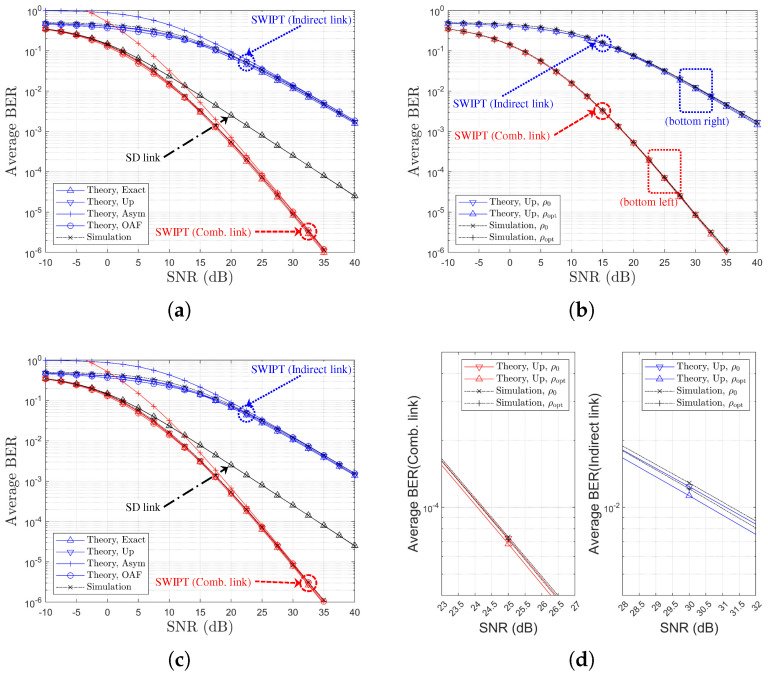
BER Performance comparison with respect to SNRs and PS optimization (SR-link-based relay selection, η=1.0, $\rho_{0}=0.5$, R=4, $N_{\mathrm{th}}=4$, Ch. Model$\;=\left[1,1,X\right]$): (**a**) $\rho_{r}=\rho_{0}$ (both links), (**b**) $\rho_{r}=\rho_{0}$ vs. $\rho_{r}=\rho_{{\mathrm{opt}}_{r}}$ (both links), (**c**) $\rho_{r}=\rho_{{\mathrm{opt}}_{r}}$ (both links), and (**d**) $\rho_{r}=\rho_{0}$ vs. $\rho_{r}=\rho_{{\mathrm{opt}}_{r}}$ (separate link).

Table 2 and Table 3 provide references between figure legends and the corresponding theoretical formulations for SR- and RD-based relay selection, respectively. Figure 3, Figure 4, Figure 5 and Figure 6 display the BER variation with respect to SNR and power splitting ratio $\rho_{r}$ under different values of $N_{\mathrm{th}}$.

From Figure 3a,c, it is evident that the indirect-only path suffers from poorer performance due to limited relay-side energy, while the combined path outperforms the direct link alone across all SNRs. The theoretical curves (‘Theory,Exact’, ‘Theory,Up’, ‘Theory,OAF’) align closely with simulated results, and the ‘Theory,Asym’ case effectively captures the high-SNR behavior. Figure 3b,d also illustrate that the BER improvements from power splitting optimization gains of approximately 1.5 dB and 0.5 dB are observed for the indirect and combined links, respectively. Finally, an increase in $N_{\mathrm{th}}$ from 1 to 4 (i.e., moving to Figure 6) results in reduced relay selection precision, reflecting a decline in the achievable diversity order.

**Figure 7:**
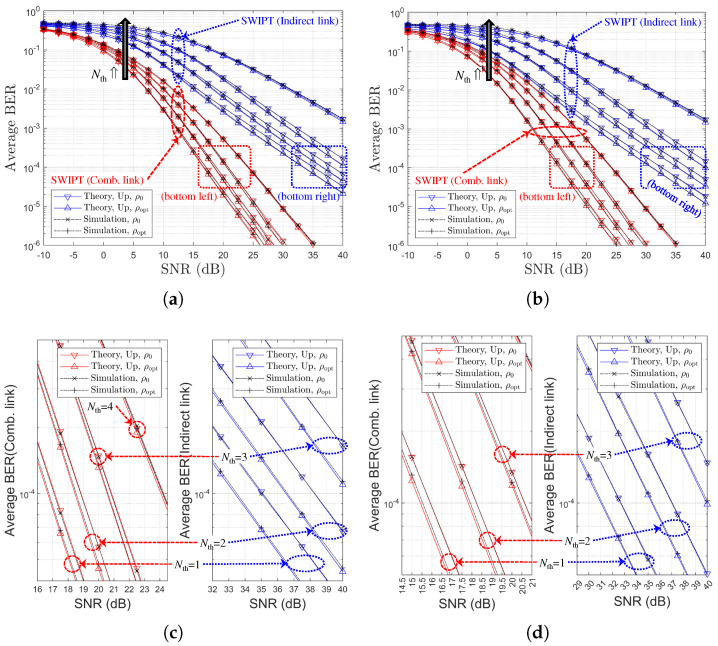
BER Performance comparison with respect to SNR, PS optimization, $N_{\mathrm{th}}$, and two channel environments (SR-link-based relay selection, η=1.0, $\rho_{r}=\rho_{0}=0.5$ or $\rho_{r}=\rho_{{\mathrm{opt}}_{r}}$, R=4 & $N_{\mathrm{th}}\in \left\{1,2,3,4\right\}$): (**a**) Ch. Model$\;=\left[1,1,X\right]$ (both links), (**b**) Ch. Model$\;=\left[1,X,1\right]$ (both links), (**c**) Ch. Model$\;=\left[1,1,X\right]$ (separate link), and (**d**) Ch. Model$\;=\left[1,X,1\right]$ (separate link).

Figure 7 presents the BER performance comparison for different values of $N_{\mathrm{th}}$, $\rho_{r}\in \left\{\rho_{0},\rho_{{\mathrm{opt}}_{r}}\right\}$, and various channel environments. It is noted that, in Figure 7a,b, the black arrows represent an increase in $N_{\mathrm{th}}$. Figure 7 shows that the PS optimization gain is maintained for the indirect link regardless of $N_{\mathrm{th}}$. However, for the combined link, the PS optimization gain diminishes as $N_{\mathrm{th}}$ increases. From Figure 7, it is observed that the selection diversity gain manifests not as a diversity order gain but rather as an SNR gain, in contrast to the general (non-SWIPT) OAF scheme discussed in [20,33]. Note that for the two different channel models, relay selection is performed based on the SR link SNRs. Therefore, under Ch. Model$\;=\left[1,X,1\right]$, a better SR link can be selected, leading to more efficient energy harvesting, which subsequently improves the RD link. Consequently, it can be concluded that Ch. Model$\;=\left[1,X,1\right]$ provides better BER performance when employing an SR link-based relay selection method.

**Figure 8:**
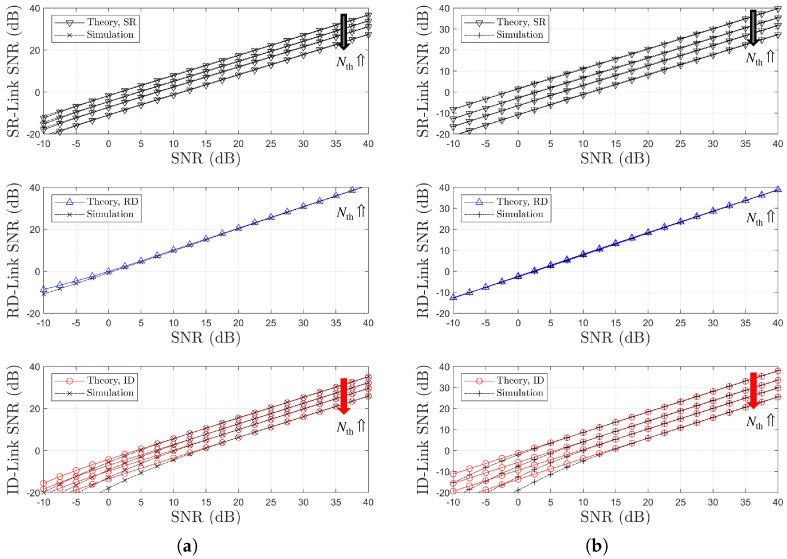
Link SNR comparison with respect to SNR (SR-link-based relay selection, η=1.0, $\rho_{r}=\rho_{{\mathrm{opt}}_{r}}$, R=4 & $N_{\mathrm{th}}\in \left\{1,2,3,4\right\}$): (**a**) Ch. Model$\;=\left[1,1,X\right]$ and (**b**) Ch. Model$\;=\left[1,X,1\right]$.

Figure 8 illustrates the variations in link SNR performance with respect to $N_{\mathrm{th}}$ resulting from the optimization of the power splitting ratio ρ. It is noted that, in Figure 8a,b, the black and red arrows represent an increase in $N_{\mathrm{th}}$. It can be observed that as $N_{\mathrm{th}}$ increases, a less favorable relay with weaker link conditions is selected. Consequently, the performance gap between the two links becomes more pronounced. These results indicate that the selection diversity gain typically associated with OAF relaying is not evident, even when the optimal power splitting ratio $\rho_{r}=\rho_{{\mathrm{opt}}_{r}}$ is employed. Note that for two channel models, the relay selection is performed based on the SR link CSIs using (17) and (19), resulting in a decrease in the SR link SNR as $N_{\mathrm{th}}$ increases. In contrast, the RD link SNR remains constant.

### 5.2. SWIPT OAF Relaying System Using P-CSIs Based on RD Links

Table 3 summarizes the relationship between the legend descriptions, equation numbers, and symbols used in the figures corresponding to relay selection based on RD links. Accordingly, in this subsection, we derive the theoretical results for the equations listed in Table 3.

**Figure 9:**
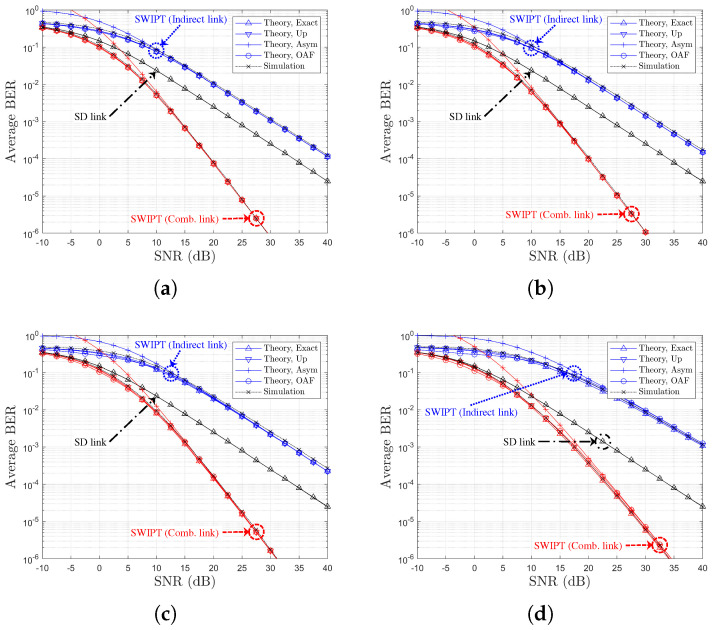
BER performance comparison with respect to SNRs and $N_{\mathrm{th}}$ (RD-link-based relay selection, η=1.0, $\rho_{r}=\rho_{{\mathrm{opt}}_{r}}$, R=4 & $N_{\mathrm{th}}\in \left\{1,2,3,4\right\}$, Ch. Model$\;=\left[1,1,X\right]$): (**a**) $N_{\mathrm{th}}=1$, (**b**) $N_{\mathrm{th}}=2$, (**c**) $N_{\mathrm{th}}=3$, and (**d**) $N_{\mathrm{th}}=4$.

Figure 9 presents the BER performance as functions of the direct link SNR for various values of $N_{\mathrm{th}}$. In this figure, the theoretical results labeled as ‘Theory,Exact’, ‘Theory,Up’, and ‘Theory,OAF’ exhibit excellent agreement with the simulation results across all SNR values. In particular, ‘Theory,Asym’ accurately captures the asymptotic BER behavior in the high-SNR regime, although the SNR threshold at which the theoretical and simulation results begin to converge increases with $N_{\mathrm{th}}$. It should be noted that in Figure 9c,d, discrepancies are observed between the theoretical and simulation results. These discrepancies primarily arise because the theoretical predictions for $\rho_{{\mathrm{opt}}_{r}}$ are derived from asymptotic BER expressions.

**Figure 10:**
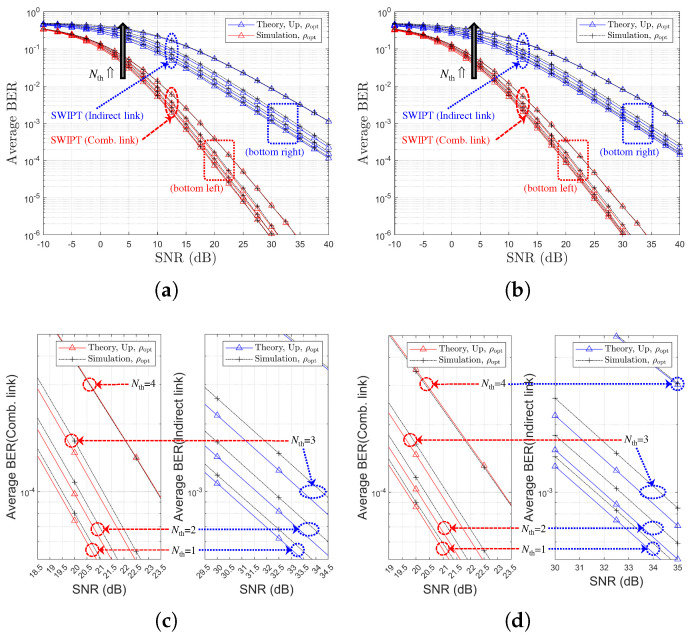
BER performance comparison with respect to SNRs, $N_{\mathrm{th}}$, and two channel environments (RD-link-based relay selection, η=1.0, $\rho_{r}=\rho_{{\mathrm{opt}}_{r}}$, R=4 & $N_{\mathrm{th}}\in \left\{1,2,3,4\right\}$): (**a**) Ch. Model$\;=\left[1,1,X\right]$ (both links), (**b**) Ch. Model$\;=\left[1,X,1\right]$ (both links), (**c**) Ch. Model$\;=\left[1,1,X\right]$ (separate link), and (**d**) Ch. Model$\;=\left[1,X,1\right]$ (separate link).

**Figure 11:**
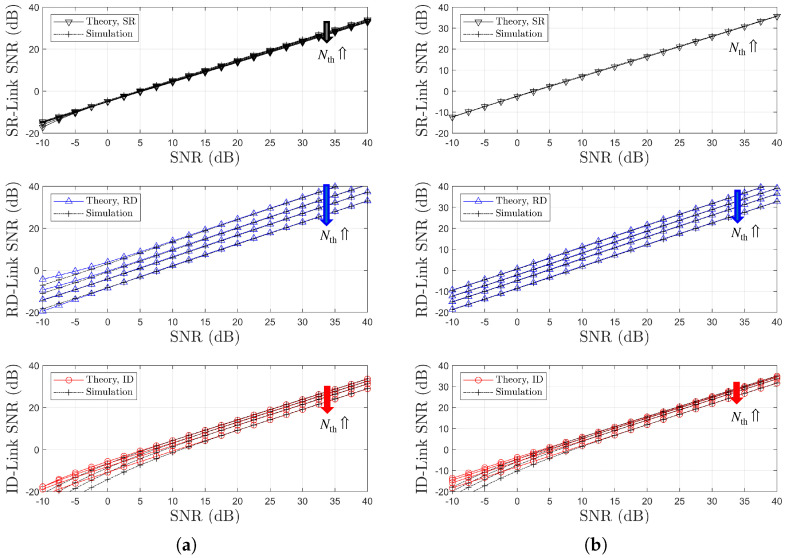
Link SNR Comparison with respect to SNR (RD-link-based relay selection, η=1.0, $\rho_{r}=\rho_{{\mathrm{opt}}_{r}}$, R=4 & $N_{\mathrm{th}}\in \left\{1,2,3,4\right\}$): (**a**) Ch. Model$\;=\left[1,1,X\right]$ and (**b**) Ch. Model$\;=\left[1,X,1\right]$.

For different two channel models, Figure 10 and Figure 11 illustrate the BER performance comparison and the link SNR performance comparison, respectively, with respect to $N_{\mathrm{th}}$. It is noted that, in Figure 10a,b, the black arrows represent an increase in $N_{\mathrm{th}}$. It is noted that, in Figure 11a,b, the black, blue, and red arrows represent an increase in $N_{\mathrm{th}}$. Since the relay selection is based on RD link information, an increase in $N_{\mathrm{th}}$ leads to a decrease in the RD link SNR, as shown in Figure 11. In contrast, the SR link SNR remains nearly constant regardless of $N_{\mathrm{th}}$. This trend is the opposite of that observed in the SR-link-based relay selection case. It is confirmed that the BER performance deteriorates as $N_{\mathrm{th}}$ increases, which manifests as an SNR loss (see Figure 10a,b), while no significant degradation in diversity order is observed. The accuracy of the proposed analytical method is also validated through this comparison.

Note that for the two different channel models, the relay selection method is based on the RD link SNRs. Under ‘Ch. Model$\;=\left[1,1,X\right]$’, rather than ‘Ch. Model$\;=\left[1,X,1\right]$’, a better RD link can be selected more frequently, resulting in an SNR gain for the indirect link. Therefore, in the RD-link-based relay selection method, it can be confirmed that $\left[1,1,X\right]$ corresponds to a better channel environment than $\left[1,X,1\right]$. This trend is consistent with the relationship observed in Figure 7.

### 5.3. Performance Comparison by Relay Selection Method: SR Links vs. RD Links

**Figure 12:**
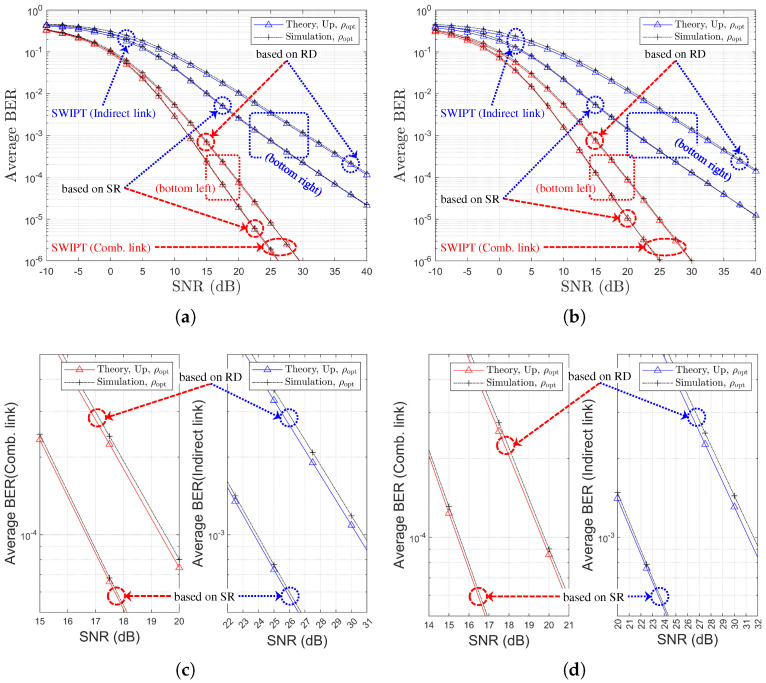
BER performance comparison with respect to SNRs, channel environments, and relay selection strategies (η=1.0, $\rho_{r}=\rho_{{\mathrm{opt}}_{r}}$, R=4 & $N_{\mathrm{th}}=1$): (**a**) Ch. Model$\;=\left[1,1,X\right]$ (both links), (**b**) Ch. Model$\;=\left[1,X,1\right]$ (both links), (**c**) Ch. Model$\;=\left[1,1,X\right]$ (separate link), and (**d**) Ch. Model$\;=\left[1,X,1\right]$ (separate link).

**Figure 13:**
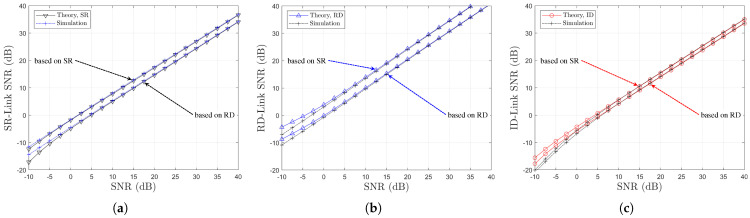
Link SNR comparison with respect to SNRs and relay selection strategies (η=1.0, $\rho_{r}=\rho_{{\mathrm{opt}}_{r}}$, R=4 & $N_{\mathrm{th}}=1$, Ch. Model$\;=\left[1,1,X\right]$): (**a**) SR-Link, (**b**) RD-Link, and (**c**) Indirect Link.

**Figure 14:**
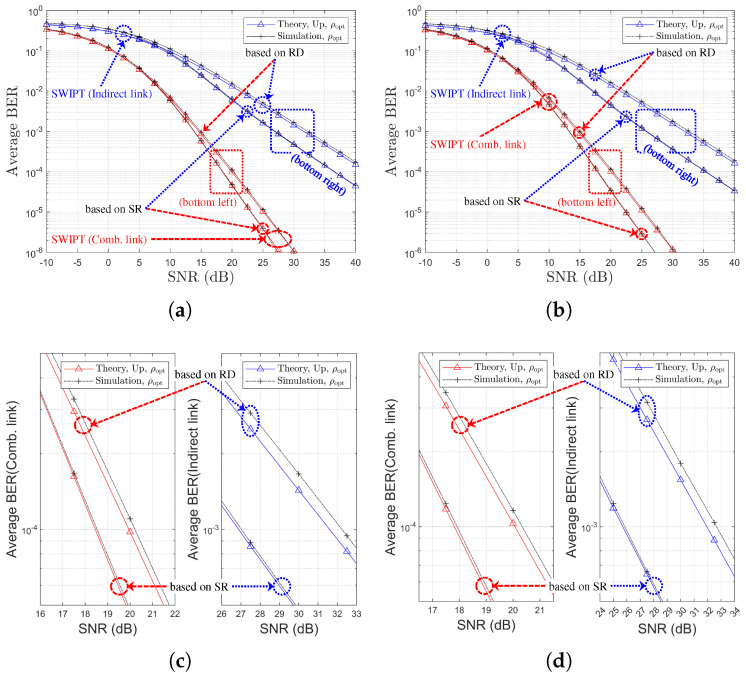
BER performance comparison with respect to SNRs, channel environments, and relay selection strategies (η=1.0, $\rho_{r}=\rho_{{\mathrm{opt}}_{r}}$, R=4 & $N_{\mathrm{th}}=2$): (**a**) Ch. Model$\;=\left[1,1,X\right]$ (both links), (**b**) Ch. Model$\;=\left[1,X,1\right]$ (both links), (**c**) Ch. Model$\;=\left[1,1,X\right]$ (separate link), and (**d**) Ch. Model$\;=\left[1,X,1\right]$ (separate link).

**Figure 15:**
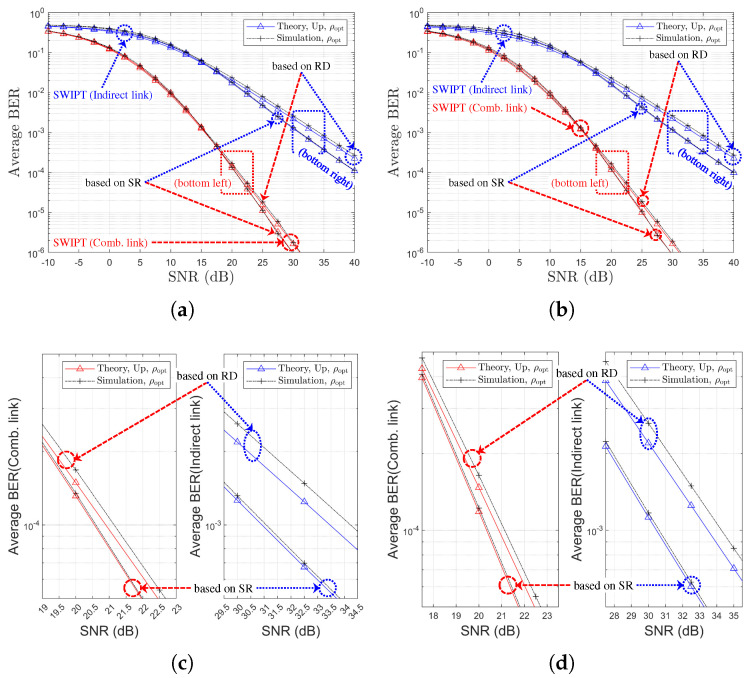
BER performance comparison with respect to SNRs, channel environments, and relay selection strategies (η=1.0, $\rho_{r}=\rho_{{\mathrm{opt}}_{r}}$, R=4 & $N_{\mathrm{th}}=3$): (**a**) Ch. Model$\;=\left[1,1,X\right]$ (both links), (**b**) Ch. Model$\;=\left[1,X,1\right]$ (both links), (**c**) Ch. Model$\;=\left[1,1,X\right]$ (separate link), and (**d**) Ch. Model$\;=\left[1,X,1\right]$ (separate link).

By comparing Figure 7 to Figure 10, we can determine which relay selection method is superior in the given channel environment. At both $\left[1,1,X\right]$ and $\left[1,X,1\right]$ environments, the SR-link-based method is at least the same or better for all $N_{\mathrm{th}}\in \left\{1,2,3,4\right\}$. Since SWIPT relay does not have its own power source, even if the optimal relay is selected based on the RD link, this selection diversity cannot be fully utilized because EH is performed based on the SR link. Figure 12, Figure 13, Figure 14 and Figure 15 illustrates the performance comparison between the two selection schemes.

Figure 12 illustrates the average BER performance of SWIPT OAF relaying systems under two partial CSI-based relay selection strategies: one based on SR links and the other on RD links. The results are presented for both the indirect link (in blue) and the combined link (in red). It is clearly observed that the SR-based relay selection yields significantly better BER performance compared to the RD-based selection. This finding implies that selecting a relay with a strong SR channel contributes more effectively to improving the end-to-end link quality. In contrast, the RD-based selection results in higher BER, indicating that a strong RD link alone does not guarantee improved performance for either the indirect or combined links.

Figure 13 presents the average link SNR performance of the SWIPT OAF relaying system under two partial CSI-based relay selection strategies. In Figure 13a, the SR-link SNR is maximized under SR-based selection, whereas RD-based selection yields significantly lower SR-link performance due to the exclusion of SR-link quality from the selection metric. Figure 13c indicates that the ID-link SNR, representing the end-to-end performance, is higher under SR-based selection, consistent with the better BER performance observed in Figure 12. Figure 14 and Figure 15 show the BER performance comparison for the two partial CSI-based relay selection strategies, corresponding to $N_{\mathrm{th}}=2$ and $N_{\mathrm{th}}=3$, respectively.

From Figure 12, Figure 14 and Figure 15, it can be observed that as $N_{\mathrm{th}}$ increases, the performance difference between the two selection techniques diminishes. Note that Table 4 summarizes the SNR gain achieved by the SR-based relay selection method compared to the RD-based scheme.

These results confirm that selecting relays based on SR-link CSI offers superior end-to-end performance, despite a slight degradation in RD-link quality. In all cases, the theoretical results exhibit close agreement with the simulation outcomes, thereby validating the accuracy of the analytical derivations.

## 6. Conclusions

This paper presented an exact and asymptotic error performance analysis of SWIPT OAF relaying systems over Rayleigh fading channels. Building upon our previous work, which modeled SWIPT AF relays as generalized AF systems, we extended this analytical framework to encompass the SWIPT OAF scenario. This novel interpretation enables the derivation of simplified closed-form SER and BER expressions, providing valuable insights into the impact of the SR and RD link qualities and the power splitting ratio ρ on system performance.

Our analysis revealed that SR-based relay selection generally offers superior performance compared to RD-based selection under partial CSI. Extensive simulations confirmed the accuracy of the analytical results and demonstrated that the proposed generalized non-SWIPT OAF model effectively approximates the performance of actual SWIPT OAF systems across various SNR conditions and system configurations.

Overall, the proposed analytical framework significantly simplifies the performance evaluation and design of SWIPT OAF relaying systems, offering a practical tool for system optimization and deployment.

While the proposed protocol provides performance advantages in terms of analytical tractability and practical relevance, it also presents certain limitations and opportunities for future enhancement. In particular, as illustrated in Figure 2, the OAF protocol requires additional signaling overhead associated with relay selection under partial CSI. However, this overhead can be substantially mitigated if the relay selection is integrated into the initial call setup procedures commonly executed before data transmission. Considering this reduction in practical overhead and the significant BER improvement demonstrated by the proposed method, we believe the OAF-based approach remains highly applicable and promising for real-world implementations.

Nonetheless, the proposed approach lays a foundation for further research in energy-efficient relay selection, integration with mobility-aware systems, and extensions to more complex fading environments or network conditions. These aspects are addressed in more detail in the future work directions outlined below.

### Future Work

Several promising research directions arise from this study:

- Nonlinear EH Modeling and Efficiency Variability: Practical energy harvesting (EH) circuits often exhibit nonlinear characteristics and time-varying conversion efficiencies due to hardware limitations, temperature, and other environmental factors. Incorporating nonlinear EH models, as discussed in [41], and analyzing the impact of efficiency variability can enhance the accuracy and robustness of performance evaluations and system design for real-world deployment.
- Machine-Learning-Based PS Optimization: Adaptive power splitting (PS) control using machine learning techniques—such as deep reinforcement learning and predictive modeling [42]—could improve system adaptability and energy efficiency under dynamic wireless environments with partial CSI constraints.
- Advanced Optimization Algorithms: Employing advanced nonlinear optimization methods, including those surveyed in [40], may provide more efficient solutions to nonconvex optimization problems typically encountered in SWIPT OAF relaying systems.
- Analysis under Generalized Fading: Extending the current analytical framework to incorporate more general channel models, such as Rician, Nakagami-*m*, or κ-μ fading, may provide valuable insights into the performance of SWIPT systems under line-of-sight (LoS) or composite fading conditions prevalent in practical deployment scenarios.
- Cross-Layer and System-Level Design: Integrating higher-layer design aspects such as MAC-layer scheduling, delay constraints, and buffer management could lead to a more comprehensive and practically viable design for SWIPT OAF relay networks in next-generation IoT and wireless systems.

These directions are expected to improve the efficiency, resilience, and real-world applicability of SWIPT OAF relaying systems in future wireless communication infrastructures.

## Figures and Tables

**Table 1:** Channel Models for SWIPT OAF Relaying System.

Ch. Model	Link	Channel Power	*R*
$\left[1,1,X\right]$ ^1^	SD	$\Omega_{0}=1$	4
	SR	${\left.\Omega_{r}\right|}_{r=1}^{R}=1$	
	RD	$\Omega_{R+r}\in \left\{1/2,1,2,4\right\}$	
$\left[1,X,1\right]$ ^2^	SD	$\Omega_{0}=1$	4
	SR	$\Omega_{r}\in \left\{1/2,1,2,4\right\}$	
	RD	${\left.\Omega_{R+r}\right|}_{r=1}^{R}=1$	

^1^ When $X\left(=\Omega_{R+r}\right)\in \left\{1/2,1,2,4\right\}$, $\left[1,1,X\right]=\left[\Omega_{0},\Omega_{r},\Omega_{R+r}\right]$ indicates a channel environment with different RD link channel powers. ^2^ When $X\left(=\Omega_{r}\right)\in \left\{1/2,1,2,4\right\}$, $\left[1,X,1\right]=\left[\Omega_{0},\Omega_{r},\Omega_{R+r}\right]$ indicates a channel environment with different SR link channel powers.

**Table 2:** Legend description for SWIPT OAF relaying systems based on P-CSIs of SR links.

Legend	Symbol	Equation(s)	Remarks
Theory,Exact	$P_{S,\mathrm{id}}$, $P_{S,\mathrm{cb}}$	(26)	Exact MGF
Theory,Up	$P_{S,\mathrm{id}}^{\mathrm{Up}}$, $P_{S,\mathrm{cb}}^{\mathrm{Up}}$	(27)	Upper Bound MGF
Theory,Asym	$P_{B,\mathrm{id}}^{\mathrm{Asym}}$, $P_{B,\mathrm{cb}}^{\mathrm{Asym}}$	(28), (29)	Asymptotic Bound
Theory,OAF	$P_{B,\mathrm{id}}^{\mathrm{OAF}}$, $P_{B,\mathrm{cb}}^{\mathrm{OAF}}$	(39), (40)	OAF Approximation
Theory,SR	${\bar{\gamma }}_{N_{\max}}=\left({\bar{\gamma }}_{\mathrm{sr}}^{N_{\max}}\right)$	(30)	Exact
Theory,RD	${\bar{\gamma }}_{\mathrm{rd}}^{N_{\max}}$	(32)	Exact
Theory,ID	${\bar{\gamma }}_{\mathrm{id}}^{N_{\max}}$	(33)	High SNR Approximation

**Table 3:** Legend description for SWIPT OAF relaying systems based on P-CSIs of RD links.

Legend	Symbol	Equation(s)	Remarks
Theory,Exact	$P_{S,\mathrm{id}}$, $P_{S,\mathrm{cb}}$	(52)	Exact MGF
Theory,Up	$P_{S,\mathrm{id}}^{\mathrm{Up}}$, $P_{S,\mathrm{cb}}^{\mathrm{Up}}$	(53)	Upper Bound MGF
Theory,Asym	$P_{B,\mathrm{id}}^{\mathrm{Asym}}$, $P_{B,\mathrm{cb}}^{\mathrm{Asym}}$	(54), (55)	Asymptotic Bound
Theory,OAF	$P_{B,\mathrm{id}}^{\mathrm{OAF}}$, $P_{B,\mathrm{cb}}^{\mathrm{OAF}}$	(63), (64)	OAF Approximation
Theory,SR	${\bar{\gamma }}_{N_{\max}}=\left({\bar{\gamma }}_{\mathrm{sr}}^{N_{\max}}\right)$	(56)	Exact
Theory,RD	${\bar{\gamma }}_{\mathrm{rd}}^{N_{\max}}$	(58)	Exact
Theory,ID	${\bar{\gamma }}_{\mathrm{id}}^{N_{\max}}$	(59)	High SNR Approximation

**Table 4:** SNR gain (dB) according to relay selection criteria.

Ch. Model	$N_{\mathrm{th}}$	Gain for Comb. Link ^1^	Gain for Indirect Link ^2^	Figures
$\left[1,1,X\right]$	1	>2.5	>6.5	Figure 12a,c
	2	>1.5	>5.0	Figure 14a,c
	3	>0.5	>3.0	Figure 15a,c
$\left[1,X,1\right]$	1	>4.0	>9.5	Figure 12b,d
	2	>2.5	>6.5	Figure 14b,d
	3	>0.5	>3.5	Figure 15b,d

^1^ SNR gain (dB) for the combined link, based on simulation results at $\mathrm{BER}=10^{-4}$. ^2^ SNR gain (dB) for the indirect link, based on simulation results at $\mathrm{BER}=10^{-3}$.

## Data Availability

Data are contained within the article.

## Abbreviations

The following abbreviations are used in this manuscript:

AF	amplify-and-forward
AWGN	additive white Gaussian noise
BER	bit error rate
BPSK	binary phase shift keying
CDF	cumulative distribution function
CSI	channel state information
DF	decode-and-forward
D2D	Device-to-Device
EH	energy harvesting
IP	information processing
MGF	moment-generating function
OAF	opportunistic amplify-and-forward
PDF	probability density function
PS	power splitting
PSF	power splitting factor
RD	relay-to-destination
RF	radio frequency
SD	source-to-destination
SRD	source-relay-destination
SER	symbol error rate
SNR	signal-to-noise ratio
SR	source-to-relay
SWIPT	simultaneous wireless information and power transfer
TS	time splitting
WPCN	wireless powered communication network

## Appendix A. Generalized Interpretation of SWIPT AF Relaying as Conventional AF Relaying [36]

### Appendix A.1. MGF and Upper Bounded Exprassions

Consider the joint PDF of the random variable pair $\left\{\gamma_{r},\beta_{r}\right\}$ defined in (9), given by

(A1) $$\begin{array}{cc} f_{\gamma_{r},\beta_{r}}\left(x,y\right)\; & =\frac{1}{{\bar{\gamma }}_{r}}\exp\left(-\frac{x}{{\bar{\gamma }}_{r}}\right)\frac{1}{{\bar{\beta }}_{r}}\exp\left(-\frac{y}{{\bar{\beta }}_{r}}\right)\;u\left(x\right)\;u\left(y\right) \end{array}$$

where $u\left(\cdot \right)$ denotes the unit step function [43]. The moment generating function (MGF) of $\gamma_{{\mathrm{id}}_{r}}$, where $\gamma_{{\mathrm{id}}_{r}}=\frac{\gamma_{r}\beta_{r}}{\beta_{r}+1}$ as in (8), is derived in [36] as

(A2) $$\begin{array}{cc} M_{\mathrm{id}}\left(s|{\bar{\gamma }}_{r},{\bar{\beta }}_{r}\right)\; & =\int_{y=0}^{\infty }\left[\int_{x=0}^{\infty }\frac{1}{{\bar{\gamma }}_{r}}\;e^{-\frac{xy}{y+1}s}\;e^{-\frac{x}{{\bar{\gamma }}_{r}}}\;dx\right]\frac{1}{{\bar{\beta }}_{r}}\;e^{-\frac{y}{{\bar{\beta }}_{r}}}\;dy\\ \; & =\frac{1}{a}+\frac{\left(a-1\right)}{a}\cdot \frac{1}{\alpha }\;\exp\left(\frac{1}{\alpha }\right)\;E_{1}\left(\frac{1}{\alpha }\right) \end{array}$$

where $\alpha ={\bar{\beta }}_{r}\;a$ and $a={\bar{\gamma }}_{r}\;s+1$ [37,38,44]. Moreover, the MGF in (A2) admits an upper bound given by [38]

(A3) $$\begin{array}{cc} M_{\mathrm{id}}\left(s|{\bar{\gamma }}_{r},{\bar{\beta }}_{r}\right)\; & <\frac{1}{{\bar{\gamma }}_{r}\;s+1}+\frac{{\bar{\gamma }}_{r}\;s}{{\bar{\gamma }}_{r}\;s+1}\cdot \frac{\ln\left[{\bar{\beta }}_{r}\left({\bar{\gamma }}_{r}\;s+1\right)+1\right]}{{\bar{\beta }}_{r}\left({\bar{\gamma }}_{r}\;s+1\right)}\\ \; & =M_{\mathrm{id}}^{\mathrm{Up}}\left(s|{\bar{\gamma }}_{r},{\bar{\beta }}_{r}\right). \end{array}$$

### Appendix A.2. Asymptotic BER Expression

For BPSK modulation, the average BER of the SD link is given by

(A4) $$\begin{array}{c} P_{B,\mathrm{sd}}=\int_{0}^{\infty }Q\left(\sqrt{2x}\right)\frac{1}{{\bar{\gamma }}_{\mathrm{sd}}}\exp\left(-\frac{x}{{\bar{\gamma }}_{\mathrm{sd}}}\right)dx \end{array}$$

where ${\bar{\gamma }}_{\mathrm{sd}}$ denotes the average SNR of the SD link.

Then, $P_{B,\mathrm{sd}}$ can be expressed in the general asymptotic form [39] as

(A5) $$\begin{array}{c} P_{B,\mathrm{sd}}\approx \frac{1}{4{\bar{\gamma }}_{\mathrm{sd}}}. \end{array}$$

The derivation of (A5) from (A4) is based on approximating $Q\left(\sqrt{2x}\right)$ as $\exp\left(-4x\right)$ for large *x* [36]. This yields

(A6) $$\begin{array}{c} P_{B,\mathrm{sd}}\approx \int_{0}^{\infty }e^{-4x}\frac{1}{{\bar{\gamma }}_{\mathrm{sd}}}e^{-x/{\bar{\gamma }}_{\mathrm{sd}}}dx=\frac{1}{4{\bar{\gamma }}_{\mathrm{sd}}+1}<\frac{1}{4{\bar{\gamma }}_{\mathrm{sd}}}. \end{array}$$

It should be noted that the asymptotic expression in (A5) is valid for ${\bar{\gamma }}_{\mathrm{sd}}\gg 1$, i.e., under the high-SNR approximation [39].

With the same argument, using $M_{\mathrm{id}}\left(s|{\bar{\gamma }}_{r},{\bar{\beta }}_{r}\right)$ in (A2), the asymptotic BER $P_{B,{\mathrm{id}}_{r}}$ can be written and bounded as follows:

(A7) $$\begin{array}{cc} P_{B,{\mathrm{id}}_{r}}\; & \approx \int_{y=0}^{\infty }\left[\int_{x=0}^{\infty }\frac{1}{{\bar{\gamma }}_{r}}e^{-\frac{xy}{y+1}4}e^{-\frac{x}{{\bar{\gamma }}_{r}}}dx\right]\frac{1}{{\bar{\beta }}_{r}}e^{-\frac{y}{{\bar{\beta }}_{r}}}dy={\left.M_{\mathrm{id}}\left(s|{\bar{\gamma }}_{r},{\bar{\beta }}_{r}\right)\right|}_{s=4}\\ \; & <M_{\mathrm{id}}^{\mathrm{Up}}\left(s|{\bar{\gamma }}_{r},{\bar{\beta }}_{r}\right){|}_{s=4}\\ \; & =\frac{1}{4{\bar{\gamma }}_{r}+1}+\frac{4{\bar{\gamma }}_{r}}{4{\bar{\gamma }}_{r}+1}\frac{\ln\left[{\bar{\beta }}_{r}\left(4{\bar{\gamma }}_{r}+1\right)+1\right]}{{\bar{\beta }}_{r}\left(4{\bar{\gamma }}_{r}+1\right)}=P_{B,\mathrm{id}}^{\mathrm{Asym}}\left({\bar{\gamma }}_{r},{\bar{\beta }}_{r}\right)\\ \; & =\frac{1}{4{\bar{\gamma }}_{r}+1}+\frac{1}{4{\bar{\gamma }}_{r}^{\mathrm{rd}}+1}=P_{B,\mathrm{id}}^{\mathrm{Asym}}\left({\bar{\gamma }}_{r},{\bar{\gamma }}_{r}^{\mathrm{rd}}\right). \end{array}$$

Here, ${\bar{\gamma }}_{r}^{\mathrm{rd}}$ is a function of ${\bar{\gamma }}_{r}$ and ${\bar{\beta }}_{r}$, defined as

$$\begin{array}{cc} \frac{1}{4{\bar{\gamma }}_{r}+1}+\frac{1}{4{\bar{\gamma }}_{r}^{\mathrm{rd}}+1}\; & ≜M_{\mathrm{id}}^{\mathrm{Up}}\left(s|{\bar{\gamma }}_{r},{\bar{\beta }}_{r}\right){|}_{s=4}. \end{array}$$

Furthermore, ${\bar{\gamma }}_{r}^{\mathrm{rd}}$ can be expressed as

(A8) $$\begin{array}{c} {\bar{\gamma }}_{r}^{\mathrm{rd}}=\frac{4{\bar{\gamma }}_{r}+1}{4{\bar{\gamma }}_{r}}\frac{{\bar{\beta }}_{r}{\bar{\gamma }}_{r}\frac{4{\bar{\gamma }}_{r}+1}{4{\bar{\gamma }}_{r}}}{\ln\left[4{\bar{\beta }}_{r}{\bar{\gamma }}_{r}\frac{4{\bar{\gamma }}_{r}+1}{4{\bar{\gamma }}_{r}}+1\right]}-\frac{1}{4}. \end{array}$$

Consequently, the BER $P_{B,\mathrm{id}}\left(=P_{S,\mathrm{id}}{|}_{M=2}\right)$ in (26) can be approximated asymptotically by $P_{B,\mathrm{id}}^{\mathrm{Asym}}$ in (28).

Note that, for ${\bar{\gamma }}_{r}\gg 1$, (A7) and (A8) can be asymptotically approximated, respectively, as [36]

(A9) $$\begin{array}{cc} {\bar{\gamma }}_{r}^{\mathrm{rd}} & \to \frac{{\bar{\beta }}_{r}{\bar{\gamma }}_{r}}{\ln\left(4{\bar{\beta }}_{r}{\bar{\gamma }}_{r}+1\right)}-\frac{1}{4}\\ P_{B,\mathrm{id}}^{\mathrm{Asym}}\left({\bar{\gamma }}_{r},{\bar{\beta }}_{r}\right) & \to \frac{1}{4{\bar{\gamma }}_{r}+1}+\frac{1}{4{\bar{\beta }}_{r}{\bar{\gamma }}_{r}/\ln\left(4{\bar{\beta }}_{r}{\bar{\gamma }}_{r}+1\right)}. \end{array}$$

### Appendix A.3. SWIPT AF Relaying Modeled as Conventional AF Relaying

Let $\gamma_{{\mathrm{id}}_{r}}$ in (8) be approximated as in [36]

(A10) $$\begin{array}{c} \gamma_{{\mathrm{id}}_{r}}=\frac{\gamma_{r}\beta_{r}}{\beta_{r}+1}\approx \frac{\gamma_{r}\gamma_{r}^{\mathrm{rd}}}{\gamma_{r}+\gamma_{r}^{\mathrm{rd}}}≃\min\left\{\gamma_{r},\gamma_{r}^{\mathrm{rd}}\right\}=\gamma_{r}^{\mathrm{OAF}} \end{array}$$

where the random variable (RV) $\gamma_{r}^{\mathrm{rd}}$ denotes the modified RD link SNR, whose PDF is given by $f_{\gamma_{r}^{\mathrm{rd}}}\left(x\right)=\frac{1}{{\bar{\gamma }}_{r}^{\mathrm{rd}}}\exp\left(-\frac{x}{{\bar{\gamma }}_{r}^{\mathrm{rd}}}\right)u\left(x\right)$, and ${\bar{\gamma }}_{r}^{\mathrm{rd}}$ is defined in (A8). Then, the PDF of $\gamma_{r}^{\mathrm{OAF}}$ can be expressed as

(A11) $$f_{\gamma_{r}^{\mathrm{OAF}}}\left(x\right)=\frac{1}{{\bar{\gamma }}_{r}^{\mathrm{OAF}}}\exp\left(-\frac{x}{{\bar{\gamma }}_{r}^{\mathrm{OAF}}}\right)u\left(x\right)$$

where ${\bar{\gamma }}_{r}^{\mathrm{OAF}}={\left(1/{\bar{\gamma }}_{r}+1/{\bar{\gamma }}_{r}^{\mathrm{rd}}\right)}^{-1}$ [22,34]. From (A10) and (A11), the asymptotic BER bound can be expressed as

(A12) $$\begin{array}{cc} P_{B,{\mathrm{id}}_{r}}\; & \approx P_{B,{\mathrm{id}}_{r}}^{\mathrm{OAF}}<\frac{1}{4{\bar{\gamma }}_{r}^{\mathrm{OAF}}}=\frac{1}{4{\bar{\gamma }}_{r}}+\frac{1}{4{\bar{\gamma }}_{r}^{\mathrm{rd}}}. \end{array}$$

By comparing (A12) with (A7), it is confirmed that the approximation in (A10) is valid in the sense that it yields an almost identical asymptotic BER bound.

## Appendix B. *N*_th_ Order Statistic for a Set of Independent RVs

Let *Y* denote the $N_{\mathrm{th}}$ largest value among a set of independent random variables (RVs) ${\left\{X_{i}\right\}}_{i=1}^{R}$. Then, *Y* can be expressed as

(A13) $$Y=N_{\mathrm{th}}\max_{i\in \{1,2,\cdots ,R\}}\left\{X_{i}\right\}.$$

The following definitions apply:

- PDF of the RV *Y*: $f_{Y}\left(y\right)$
- CDF of the RV *Y*: $F_{Y}\left(y\right)=\mathrm{Pr}\left\{Y\leq y\right\}$

### Appendix B.1. N*_th_* Order Statistic for IID Case

For the independent and identically distributed (IID) case, the RVs $X_{i}$ share the same probability density function (PDF), expressed as

$$f_{X_{i}}\left(x\right){|}_{i=1}^{R}=f_{X}\left(x\right),\;\;\;\;i\in \left\{1,2,\cdots ,R\right\},$$

where the cumulative distribution function (CDF) is defined by $F_{X}\left(x\right)=\int_{-\infty }^{x}f_{X}\left(t\right)dt$.

Consider *Y* defined as the $N_{\mathrm{th}}$ largest value as in (A13). Then, the event Pr{Y≤y} means that among *R* RVs, at most (N−1) RVs exceed *y*, and the remaining RVs are less than or equal to *y*. Therefore, for the IID case, Pr{Y≤y} can be expressed as

(A14) FY(y)=∑k=1NRk−1Pr{Xi≤y}R−k+1Pr{Xi>y}k−1=∑k=1NRk−1FX(y)R−k+11−FX(y)k−1

and the PDF of the $N_{\mathrm{th}}$ order statistic is given by

(A15) fY(y)=RR−1N−1FX(y)R−N1−FX(y)N−1fX(y).

### Appendix B.2. N*_th_* Order Statistic for IID Rayleigh RVs

For the IID Rayleigh random variables with PDF $f_{X}\left(x\right)=\frac{1}{\bar{\gamma }}\exp\left(-\frac{x}{\bar{\gamma }}\right)u\left(x\right)$, where $\bar{\gamma }=E\left[X\right]$ is the average power, the PDF in (A15) can be expressed as

(A16) fYy=RR−1N−1∑k=0R−N(−1)kR−Nkk+N1γ¯/(k+N)exp−yγ¯/(k+N).

### Appendix B.3. N*_th_* Order Statistic for INID Case

The main objective is to derive the PDF of the RV *Y* defined by (A13). Note that among *R* RVs, each RV can be selected as the $N_{\mathrm{th}}$ maximum.

When the *i*th RV $X_{i}$ is selected as the $N_{\mathrm{th}}$ maximum, we denote the selection probability by $p_{X_{i}}\left(y\right)$. Then, the PDF of *Y* can be written as

(A17) $$f_{Y}\left(y\right)=\sum_{i=1}^{R}f_{X_{i}}\left(y\right)\;p_{X_{i}}\left(y\right).$$

To derive the selection probability $p_{X_{i}}\left(y\right)$, define the set of indices of the non-selected RVs as

$$S_{i}=\left\{1,2,\cdots ,R\right\}\setminus \left\{i\right\}.$$

The subsets of $S_{i}$ containing exactly (N−1) elements are denoted by

(A18) SiN−1,j=λi,1N−1,j,⋯,λi,mN−1,j,⋯,λi,N−1N−1,j,   j=1,2,⋯,R−1N−1,

where each $S_{i}^{N-1,j}$ has (N−1) elements, and the index *j* denotes the *j*th subset when the subsets are arranged in ascending order based on the sum of their elements.

Note that the total number of subsets having (N−1) elements is given by

R−1N−1=(R−1)!(R−N)!(N−1)!.

Also, $\lambda_{i,m}^{N-1,j}$ denotes the *m*th element of the *j*th subset containing (N−1) elements.

In addition, the complementary set of $S_{i}^{N-1,j}$ within $S_{i}$ is defined as

(A19) $$\begin{array}{cc} S_{i}^{\bar{N-1},j}\; & =S_{i}\setminus S_{i}^{N-1,j}=\left\{\lambda_{i,1}^{\bar{N-1},j},\cdots ,\lambda_{i,m}^{\bar{N-1},j},\cdots ,\lambda_{i,(R-1)-(N-1)}^{\bar{N-1},j}\right\}. \end{array}$$

Note that

$$S_{i}^{\bar{N-1},j}\cup S_{i}^{N-1,j}=S_{i},\;S_{i}^{\bar{N-1},j}\cap S_{i}^{N-1,j}=⌀,$$

where ⌀ denotes the empty set.

Let us consider that the *i*th RV ($X_{i}$) is selected as having the $N_{\mathrm{th}}$ maximum value *y*. This means that among the remaining (R−1) RVs, (N−1) RVs must have values greater than *y* (i.e., greater than the value of the selected RV $X_{i}$), and the other (R−N) RVs must have values less than or equal to *y*. Therefore, the $N_{\mathrm{th}}$ maximum selection probability of $X_{i}$ can be expressed as

(A20) pXi(y)=∑j=1R−1N−1∏p=1N−1PrXλi,pN−1,j>y∏p=1R−NPrXλi,pN−1¯,j≤y=∑j=1R−1N−1∏p=1N−11−FXλi,pN−1,j(y)∏p=1R−NFXλi,pN−1¯,j(y)

where $\mathrm{Pr}\left\{X_{i}>y\right\}=1-F_{X_{i}}\left(y\right)$ and $\mathrm{Pr}\left\{X_{i}\leq y\right\}=F_{X_{i}}\left(y\right)$.

### Appendix B.4. N*_th_* Order Statistic for INID Rayleigh RVs [33]

For independent and not identically distributed (INID) Rayleigh RVs, the PDF of $X_{i}$ is given by

(A21) $$f_{X_{i}}\left(x\right)=\frac{1}{{\bar{\gamma }}_{i}}\exp\left(-\frac{x}{{\bar{\gamma }}_{i}}\right)u\left(x\right)$$

where ${\bar{\gamma }}_{i}=E\left[X_{i}\right]$ is the mean of $X_{i}$, and the corresponding CDF is $F_{X_{i}}\left(x\right)=1-\exp\left(-\frac{x}{{\bar{\gamma }}_{i}}\right)u\left(x\right)$.

In addition, to express (A20) more tractably, we consider the set $S_{i}^{\bar{N-1},j}$ with (R−N) elements. Among the subsets of $S_{i}^{\bar{N-1},j}$, the *l*th subset having *k* elements is defined as

(A22) $$\begin{array}{cc} {\left(S_{i}^{\bar{N-1},j}\right)}^{k,l}\; & =\left\{{\left(\lambda_{i}^{\bar{N-1},j}\right)}_{1}^{k,l},\cdots ,{\left(\lambda_{i}^{\bar{N-1},j}\right)}_{m}^{k,l},\cdots ,{\left(\lambda_{i}^{\bar{N-1},j}\right)}_{k}^{k,l}\right\} \end{array}$$

where ${\left(\lambda_{i}^{\bar{N-1},j}\right)}_{m}^{k,l}$ denotes the *m*th element of the subset ${\left(S_{i}^{\bar{N-1},j}\right)}^{k,l}$.

Then, from (A20), it follows that

(A23) $$\begin{array}{cc} \prod_{p=1}^{N-1}\mathrm{Pr}\left\{X_{\lambda_{i,p}^{N-1,j}}>y\right\}\; & =\prod_{p=1}^{N-1}\exp\left(-\frac{y}{{\bar{\gamma }}_{\lambda_{i,p}^{N-1,j}}}\right)=\exp\left(-y\sum_{p=1}^{N-1}\frac{1}{{\bar{\gamma }}_{\lambda_{i,p}^{N-1,j}}}\right). \end{array}$$

Also, the binomial theorem provides

(A24) ∏p=1R−NPrXλi,pN−1¯,j≤y=∏p=1R−N1−exp−y/γ¯λi,pN−1¯,j=1+∑k=1R−N(−1)k∑l=1R−Nkexp−y∑m=1k1/γ¯λiN−1¯,jmk,l.

From (A23) and (A24), the selection probability in (A20) can be represented as

(A25) pXiy=∑j=1R−1N−1∑k=0R−N(−1)k∑l=1R−Nkexp−yBij,k,l

where

$$B_{i}^{j,k,l}=\sum_{m=1}^{N-1}1/{\bar{\gamma }}_{\lambda_{i,m}^{N-1,j}}+\sum_{m=1}^{k}1/{\bar{\gamma }}_{{\left(\lambda_{i}^{\bar{N-1},j}\right)}_{m}^{k,l}}$$

and ${\left.\sum_{m=1}^{k}\left(\cdot \right)\right|}_{k=0}=0$.

Finally, from (A21) and (A25), the PDF $f_{Y}\left(y\right)$ in (A17) can be expressed as

(A26) fY(y)=∑i=1RfXi(y)pXi(y)=∑i=1R∑j=1R−1N−1∑k=0R−N∑l=1R−Nkγ¯i′(−1)kγ¯i·1γ¯i′exp−yγ¯i′=∑⋯∑︸i,j,k,lγ¯i′(−1)kγ¯i·1γ¯i′exp−yγ¯i′

where $1/{\bar{\gamma }}_{i}^{\prime }=1/{\bar{\gamma }}_{i}+B_{i}^{j,k,l}$. For convenience, the indices *j*, *k*, and *l* in $1/{\bar{\gamma }}_{i}^{\prime }$ can be omitted. In (A26), the quadruple summation is defined as

(A27) ∑i=1R∑j=1R−1N−1∑k=0R−N∑l=1R−Nk=∑⋯∑︸i,j,k,l.

Note that (A26) also covers the IID case. By letting ${\bar{\gamma }}_{\lambda_{i,m}^{N-1,j}}={\bar{\gamma }}_{{\left(\lambda_{i}^{\bar{N-1},j}\right)}_{m}^{k,l}}=\bar{\gamma }$ and setting $B_{i}^{j,k,l}=\left(N-1+k\right)/\bar{\gamma }$, the summation $\sum_{i=1}^{R}f_{X_{i}}\left(y\right)\;p_{X_{i}}\left(y\right)$ can be rewritten as

(A28) ∑i=1RfXi(y)pXiy=∑i=1R1γ¯exp−yγ¯∑j=1R−1N−1∑k=0R−N(−1)k∑l=1R−Nkexp−yN−1+kγ¯=RR−1N−1∑k=0R−N(−1)kR−Nkγ¯exp−yN+kγ¯.

This result is identical to (A16).
